# Unilateral intranigral administration of β-sitosterol β-D-glucoside triggers pathological α-synuclein spreading and bilateral nigrostriatal dopaminergic neurodegeneration in the rat

**DOI:** 10.1186/s40478-020-00933-6

**Published:** 2020-04-22

**Authors:** Luis O. Soto-Rojas, Irma A. Martínez-Dávila, Claudia Luna-Herrera, María E. Gutierrez-Castillo, Francisco E. Lopez-Salas, Bismark Gatica-Garcia, Guadalupe Soto-Rodriguez, María Elena Bringas Tobon, Gonzalo Flores, America Padilla-Viveros, Cecilia Bañuelos, Víctor Manuel Blanco-Alvarez, José Dávila-Ayala, David Reyes-Corona, Linda Garcés-Ramírez, Oriana Hidalgo-Alegria, Fidel De La Cruz-lópez, Daniel Martinez-Fong

**Affiliations:** 1 Facultad de Estudios Superiores Iztacala, Universidad Nacional Autónoma de México, Av. de los Barrios 1, 54090 Tlalnepantla, Edo. de México Mexico; 2 Departamento de Fisiología, Escuela Nacional de Ciencias Biológicas, Instituto Politécnico Nacional, Av. Wilfrido Massieu s/n, Unidad Profesional “Adolfo López Mateos”, 07738 Ciudad de México, Mexico; 3 Departamento de Fisiología, Biofísica y Neurociencias, Centro de Investigación y de Estudios Avanzados del Instituto Politécnico Nacional, Av. Instituto Politécnico Nacional, No. 2508, 07360 Ciudad de México, Mexico; 4 Departamento de Biociencias e Ingeniería, Centro Interdisciplinario de Investigaciones y Estudios sobre Medio Ambiente y Desarrollo, Instituto Politécnico Nacional, 30 de Junio de 1520 s/n, 07340 Ciudad de México, Mexico; 5 Programa de Doctorado en Nanociencias y Nanotecnología, Av. Instituto Politécnico Nacional No. 2508, Centro de Investigación y de Estudios Avanzados, 07360 Ciudad de México, Mexico; 6 Facultad de Medicina, Benemérita Universidad Autónoma de Puebla, Calle 13 Sur 2702, Puebla 72420 Puebla, Mexico; 7 Instituto de Fisiología, Benemérita Universidad Autónoma de Puebla, 14 Sur 6301, 72570 Puebla, Puebla Mexico; 8 Coordinación General de Programas de Posgrado Multidisciplinarios, Programa de Doctorado Transdisciplinario en Desarrollo Científico y Tecnológico para la Sociedad, Av. Instituto Politécnico Nacional No. 2508, Centro de Investigación y de Estudios Avanzados, 07360 Ciudad de México, Mexico; 9 Facultad de Enfermeria, Benemérita Universidad Autónoma de Puebla, Av .25 Pte 1304, 72410 Puebla, Puebla Mexico

**Keywords:** Lewy body-like synuclein aggregations, Parkinson’s disease, BSSG, Sensorimotor alterations, Bilateral affectation, Synuclein spreading

## Abstract

The spreading and accumulation of α-synuclein and dopaminergic neurodegeneration, two hallmarks of Parkinson’s disease (PD), have been faithfully reproduced in rodent brains by chronic, oral administration of β-sitosterol β-D-glucoside (BSSG). We investigated whether a single injection of BSSG (6 μg BSSG/μL DMSO) in the left *substantia nigra* of *Wistar* rats causes the same effects. Mock DMSO injections and untreated rats formed control groups. We performed immunostainings against the pathological α-synuclein, the dopaminergic marker tyrosine hydroxylase (TH), the neuroskeleton marker β-III tubulin, the neurotensin receptor type 1 (NTSR1) as non-dopaminergic phenotype marker and Fluro-Jade C (F-J C) label for neurodegeneration. Using β-galactosidase (β-Gal) assay and active caspase-3 immunostaining, we assessed cell death mechanisms. Golgi-Cox staining was used to measure the density and types of dendritic spines of striatal medium spiny neurons. Motor and non-motor alterations were also evaluated. The study period comprised 15 to 120 days after the lesion. In the injured *substantia nigra*, BSSG caused a progressive α-synuclein aggregation and dopaminergic neurodegeneration caused by senescence and apoptosis. The α-synuclein immunoreactivity was also present within microglia cells. Decreased density of dopaminergic fibers and dendritic spines also occurred in the striatum. Remarkably, all the histopathological changes also appeared on the contralateral nigrostriatal system, and α-synuclein aggregates were present in other brain regions. Motor and non-motor behavioral alterations were progressive. Our data show that the stereotaxic BSSG administration reproduces PD α-synucleinopathy phenotype in the rat. This approach will aid in identifying the spread mechanism of α-synuclein pathology and validate anti-synucleinopathy therapies.

## Introduction

α-Synucleinopathies are neurodegenerative diseases characterized by misfolded α-synuclein aggregates, which are the major component of Lewy bodies in neurons and Lewy neurites in neuronal terminals [55, 69, 82]. Of the three main types of α-synucleinopathy, Parkinson’s disease (PD) is the most common and pure α-synucleinopathy phenotype [29]. Human genetic evidence suggests that altered α-synuclein can cause the death not only of dopaminergic neurons but also of other neuronal groups in the brain. Thus, mutations in the α-synuclein gene (SNCA), resulting in A53T, A30P, E46K, G51D, and H50Q, are known to cause familial PD [21, 82]. SNCA duplications also cause PD α-synucleinopathy by increasing normal α-synuclein levels in the midbrain [41]. Besides, epidemiological studies have associated different polymorphisms around the SNCA with the lifetime risk of sporadic PD [47, 52], possibly by increasing native α-synuclein levels.

In PD, the progression of pathological α-synuclein spreading in a prion-like manner [19, 81], schematized by the Braak stages [8], correlates with the appearance of signs and symptoms [57] and presents a major obstacle to current therapy [81]. In the prodromal phase, before any motor symptoms, pathological α-synuclein inclusions appear in olfactory and lower brainstem neurons, from where they spread to the midbrain and subcortical nuclei at the motor alteration stage. Finally, those aggregates seed α-synuclein pathology in limbic and neocortical regions, giving rise to cognitive impairments [8]. The mechanisms by which α-synuclein acts as a neurotoxic agent and spreads to the brain in PD remain, however, unknown. Nevertheless, human genetic data strongly support the α-synuclein toxicity hypothesis [11], calling for the development of simpler animal models suitable to identify α-synuclein spread mechanisms and validate new therapies.

Transgenic mice reproduce the α-synucleinopathy of human familial PD with variable results (reviewed in [30]). For instance, transgenic mice overexpressing α-synuclein rarely show dopaminergic neuron loss [53] and progressive motor impairment [26], indicating that high α-synuclein levels are not toxic in mice. The SNCA mutation A53T reproduced in mice most features of familial PD, including α-synuclein aggregates in different brain nuclei, although the motor impairments appeared only at old age [26]. In contrast, double transgenic mice bearing A30P and A53T mutations reproduced several phenotypes of PD with early-onset [39] as in PD patients [88].

Similarly, viral transduction of nigral cells has aimed to evaluate the effect of either wild type SNCA or the A30P and A53T mutations expression. Again, the results are variable and confounded by the use of different transcriptional promoters and viral vectors [30]. The majority of the studies show a correlation between the presence of α-synuclein aggregates and nigral cell loss [15, 40, 42, 45]. Unfortunately, α-synuclein aggregates have not been evaluated in other brain regions in these models, which have also not been fully characterized concerning the motor and non-motor behaviors [15, 40, 42, 45].

Studies in non-transgenic rodents have demonstrated that a single intrastriatal injection of α-synuclein preformed fibrils (PFF) can generate PD α-synucleinopathy and its intracerebral spread, but with some differences depending on the phylogenetic genus. In mice, the pathological α-synuclein aggregates spread only to brain areas that innervate the striatum of the ipsilateral side to the injection [46]. The maximum loss of dopaminergic neurons is 35% at day 180 post-injection, sufficient to cause motor deficits [46]. In rats, α-synuclein pathology is bilateral in the striatum and cortical regions at day 180 post-injection but does not affect the contralateral *substantia nigra* [56]. An early appearance of α-synuclein aggregates and dopaminergic neurodegeneration in rats has been achieved by the combination of human PFF injection with adeno-associated virus-mediated delivery of human α-synuclein into the *substantia nigra* and ventral tegmental area (VTA) [74]. It should be of interest to evaluate the impact of dopaminergic neurodegeneration on the motor and non-motor behavior in this model [74].

Exposure to environmental toxins, as a cause of sporadic PD, has gained considerable interest, since this form of the disease is more frequent than the genetic types [54]. Toxins found in the flour of washed seeds from the plant Cycad, which have been linked to the amyotrophic lateral sclerosis/parkinsonism dementia complex (ALS/PDC) in the Chamorro population of Guam island, are a classic example [12, 43]. Histopathological and behavioral impairments of ALS-PDC have been recapitulated in adult mice fed daily with washed Cycad flour for 30 days [85]. In *Sprague-Dawley* rats, the feeding with Cycad flour for at least 16 weeks triggers the loss of nigral dopaminergic neurons, motor deficits, and α-synuclein aggregates in the *substantia nigra pars compacta* (SNpc) dopaminergic neurons and striatum [65]. Interestingly, no loss of motor neurons in the spinal cord was found, as occurs in mice [72]. A faithful model for PD was developed in *Sprague-Dawley* rats chronically fed with pellets supplemented with β-sitosterol D-glucoside (BSSG), a neurotoxin isolated from Cycad [77, 78]. The chronic oral administration of BSSG (3 mg/day/5 times a week for 16 weeks) replicates the time-course and order of appearance of olfactory deficits, motor impairment, and cognitive dysfunction. Also, the chronic administration replicates the sequence in which pathological α-synuclein appears in several brain nuclei, according to the Braak stages of PD [78]. Recently, we showed that a single intranigral administration of BSSG reproduces most of the features of oral administration in less time [68]. The key findings relevant for PD were the progression of motor and non-motor alterations and the loss of dopaminergic neurons, as well as the bilateral presence of Lewy body-like synuclein aggregates in the SNpc [68]. Herein, we aim at demonstrating that a single administration of BSSG (6 μg/μL DMSO) in the left SNpc causes the spread of α-synucleinopathy and bilateral neurodegeneration of the nigrostriatal dopaminergic system [68]. We showed pathological α-synuclein aggregates in different brain nuclei by immunohistochemistry. The presynaptic effects were evaluated on markers of dopaminergic neurodegeneration and the mechanism of cell death in the SNpc, using immunodetection assays and biochemical staining, respectively. Furthermore, the postsynaptic effect was evaluated on the spine density of striatal medium spiny neurons (MSN) using Golgi-Cox staining. Also, glial cells were detected by immunostaining techniques. Likewise, we evaluated behavioral sensorimotor alterations. All assays were performed from 15 to 120 days after the injury. Our results show that BSSG promotes the appearance and spreading of pathological α-synuclein aggregates, which were the primary cause of the death of dopaminergic neurons by inducing apoptosis and possibly senescence. The resulting presynaptic and postsynaptic neurodegeneration of the nigrostriatal system elicited the parkinsonism motor deficit. Besides, our results suggest that the prion-like spreading of pathological α-synuclein aggregates could promote neurodegeneration in other brain nuclei. These features agree with the PD α-synucleinopathy phenotype, thus making the stereotaxic BSSG model attractive for the identification of α-synucleinopathy spread mechanisms, and the validation of new therapies for PD and other synucleinopathies.

## Material and methods

### Animals

The Institutional Committee for the Care and Use of Laboratory Animals of the Center for Research and Advanced Studies (Cinvestav) approved our experimental protocol #162–15 based on the Official Mexican Regulation NOM-062-ZOO-1999. Male *Wistar* rats with bodyweight between 210 and 230 g were obtained from the Unit of Animal Production and Experimentation at Cinvestav. Animals were kept under inverted light-dark 12 h cycles, 22 ± 2 °C and 60 ± 5% humidity, with access to food and water ad libitum. Animals were randomly assigned to the BSSG group (*n* = 48), with a stereotaxic infusion of 6 μg BSSG /1 μL of DMSO [68]; the mock group (stereotaxic injection of 1 μL of DMSO; *n* = 48); and the untreated (UT) group (no surgery, nor treatment; *n* = 48). Six rats of each experimental group were evaluated with two independent immunostaining methods (*n* = 3 rats per each procedure per group) and six rats with Golgi-Cox staining (*n* = 6 rats per group). These assays were performed at days 15, 30, 60, and 120 after the lesion (*n* = 12 rats for immunostaining and 24 rats for Golgi-Cox straining, per every time). Eight rats of each time point were evaluated with seven independent behavioral tests (*n* = 8 rats per group and time). The number of animals was 144, which was a minimum by the experimental design in compliance with the Guide for the Care and Use of Laboratory Animals (The National Academies Collection: Reports funded by National Institutes of Health, 2011). No animal deaths occurred during the study (Online Resource 1).

### Stereotaxic BSSG administration

Rats were submitted to general anesthesia with a mixture of 10 mg/kg xylazine and 100 mg/kg ketamine via intraperitoneal (i.p.) and placed on a stereotaxic apparatus (Stoelting; Wood Dale, IL, USA). A trepan was made to infuse 6 μg of BSSG (MedChemExpress; Monmouth Junction, NJ, USA) dissolved in 1 μL of DMSO (Sigma- Aldrich; St. Louis, MO, USA) or only DMSO, through a blunt 20-gauge dental needle in the left SNpc. The coordinates were, AP, + 2.1 mm from interaural midpoint; ML, + 2.0 mm from interparietal suture; DV, − 6.8 mm from dura mater [68]. The infusion was made by a microperfusion pump (Stoelting; Wood Dale, IL, USA) at 0.15 μL/min. The wound was sutured with silk 00 and treated with a mixture of oxytetracycline and polymyxin B (Pfizer; Toluca, Mexico) to prevent infections.

### Behavioral tests

All behavioral tests were performed from 10:00 to 14:00 h. Rats were transferred to the experimental room in cages protected from light, at least 1 h before the test to allow animals acclimatization. The surfaces and devices were cleaned with 30% ethanol, and the water for the swim cylinder was changed after each evaluation to avoid the influence of odors, substances, and temperature changes.

The vibrissae evoked forelimb placing test serves to discriminate sensorimotor asymmetry in the striatum [86]. The vibrissae were rubbed against the edge of a table to generate a forelimb response (placing the rat paw on the tabletop). Healthy animals quickly place their forelimbs on the tabletop after vibrissae stimulation [61, 68]. Ten successful forelimb placements contralateral and ipsilateral to the lesion were analyzed.

The beam walking test assesses imbalance, postural instability, and motor discoordination when rats transverse a narrow beam (1 cm wide and 2 m length at a 30° angle). The alterations were appraised as the number of claudications (errors or slips per step of the hind legs) and slowness to walk across the beam. The test was executed as previously reported [28, 68].

The cylinder test is used to evaluate locomotor asymmetry [68, 87]. Rats were placed in a transparent acrylic cylinder and video recorded. The first 20 paw contacts (ipsilateral or contralateral to the lesion or both, when the paws were used simultaneously) made by the rats over the cylinder wall were quantified. The percentage of asymmetry was expressed as the number of contacts with the ipsilateral forelimb + 1/2 of simultaneous contacts, divided by the total number of contacts [ipsilateral + contralateral + simultaneous), and multiplying the quotient by 100 [68, 87].

The open-field test evaluates locomotor activity during exploration when rats are exposed to a new environment [64]. In the present study, the rats were placed on a large square box of 60 cm width and 50 cm height per wall, where the locomotor activity was measured by an automated system (Videomex-V; Columbus Instruments; Columbus, OH, USA). The distance traveled (in centimeters) was registered for 9 min [32, 68].

The depressive-like behavior was evaluated through the forced swim test. For this test, rats are exposed to water until they acquire an immobility behavior, which reflects a failure to deal with active forms of stress coping [66, 68]. The immobility time (seconds) was registered.

The asymmetry olfactory (hyposmia) was assessed by the corridor test, as reported previously [7, 68]. Rats were acclimatized in the habituation compartment to minimize exploratory behavior. Afterward, the animals were assigned to the test compartment, where chocolate pellets were placed at a distance of 11 cm each, on both sides of the corridor floor. The test was completed when the rats made a total of 20 touches with the tip of their nose over pellets or after a maximum test time of 5 min. The percentage of asymmetric olfactory responses was evaluated by the number of contralateral touches divided by the number of contralateral + ipsilateral touches, and the quotient multiplied by 100 [68].

The alteration of working and episodic memory was assessed by the novel object recognition (NOR) test [24]. This behavioral test consists of three phases: habituation, familiarization, and evaluation. In the habituation phase, the rat is allowed freely to explore the open-field arena with no objects. In the familiarization phase, the rat explores the same arena containing two identical sample objects (A + A) for 5 min. In the evaluation phase, the animal was returned to the open field with two objects, one was identical to the sample, and the other was novel (A + B). The evaluation is carried out after a retention inter-trial interval (ITI) of 1 h to assess the working memory and 24 h to determine the episodic memory. Healthy rats spend more time exploring the novel object during the test phase [18].

### Immunostaining

Immunostaining techniques were performed according to the standard procedure described elsewhere [31, 68]. Rats were deeply anesthetized with pentobarbital (50 mg/kg of body weight, i.p.) and euthanized by transcardial perfusion of 4% paraformaldehyde in phosphate-buffered saline solution (PBS). Their brains were removed and post-fixed in 4% paraformaldehyde for 24 h followed by cryoprotection in 30% sucrose. Sections of 20 μm (mesencephalon) or 30 μm (striatum) thickness were cut on a sliding microtome (Jung Histoslide 2000R; Leica, Heidelberg, Germany). The slices were distributed consecutively in 6 wells containing tissue collecting solution (TCS; 0.2 M phosphate buffer, ethylene glycol, and glycerol) at − 20 °C, reaching a total of 12–15 serial slices per well.

Slices were permeabilized by incubation in PBS/0.3% Triton X-100 (PBS/Triton), 3 times for 5 min each, and non-specific binding sites were blocked with 10% horse serum (Invitrogen; Carlsbad, CA, USA) in PBS/Triton for 1 h at room temperature (RT). Endogenous peroxidases were eliminated by incubating the slices with 3% hydrogen peroxide in PBS/Triton and 10% methanol at RT for 10 min. The primary antibodies used for the immunohistochemistry were mouse monoclonal anti-LB-509 α-synuclein (1:500; Abcam; Cambridge, MA, USA), mouse monoclonal anti-TH (1: 1000; Sigma-Aldrich; St. Louis, MO, USA), rabbit polyclonal anti-glial fibrillary acidic protein (GFAP) as an astrocytic marker (1: 500; DakoCytomation; Glostrup, Denmark), and chicken polyclonal anti-ionized calcium-binding adapter molecule 1 (Iba1) as a microglial marker (1:1000; Abcam; Cambridge, UK). All primary antibodies were incubated at 4 °C overnight. In the case of the anti-LB-509 α-synuclein antibody, a prior incubation with 80% formic acid for 20 min was done to evidence pathological aggregates of α-synuclein [4]. The secondary antibodies used in these assays were biotinylated horse anti-mouse IgG (1: 300; Vector Laboratories; Burlingame, CA, USA), horseradish peroxidase (HRP) donkey anti-rabbit IgG (1: 500; Zymed; Cambridge, MA, USA), or donkey anti-chicken IgG (1:500; Jackson ImmunoResearch; Palo Alto, CA, USA). Secondary antibodies were incubated for 1 h at RT. Immunohistochemical staining was developed using the ABC Kit (1,10; Vector Laboratories; Burlingame, CA, USA) and 3′3-diaminobenzidine (DAB; Sigma-Aldrich; St. Louis, MO, USA). Some tissues were counterstained with cresyl violet (Sigma-Aldrich; St. Louis, MO, USA) to delimit the mesencephalic nuclei.

Cellular senescence was assessed using β-Galactosidase staining before the immunohistochemistry, as reported elsewhere [16]. Briefly, tissues were washed in PBS and incubated at 37 °C overnight with X-Gal working solution, consisting of a 1:40 dilution of the X-Gal stock solution (5 mM potassium ferrocyanide crystalline, 5 mM potassium ferricyanide trihydrate, and 2 mM magnesium chloride dissolved in PBS) in X-Gal dilution buffer (4% SA-β-Gal dissolved in dimethylformamide; Sigma-Aldrich; St. Louis, MO, USA). The brain slices were washed 3 times for 5 min in PBS and mounted on slides using Entellan resin (Merck, KGaA; Darmstadt, Germany), and observed with a light Leica DMIRE2 microscope equipped with 5x, 20x, 40x, and 63x (oil immersion) objectives (Leica Microsystems; Nussloch, Germany).

The area density for immunohistochemical staining of pathological α-synuclein aggregates was measured by ImageJ software v.1.46r (The National Institutes of Health; Bethesda, MD) in the injured and control sides of the SNpc, striatum, and cortex. The measurement was made on images taken with a 40x objective of the central zone of the SNpc, dorsolateral striatum (DL- striatum), and in the primary motor cortex (M1) in three anatomic levels (one caudal, one medial, and one rostral) of each nucleus per rat (*n* = 3 independent rats per group and time). A similar procedure was followed to measure the area density of β-Gal staining in the SNpc. The mean value calculated from the quantification in the three levels per nucleus and per rat was the final measurement.

For the double immunofluorescence assays, the primary antibodies used were rabbit polyclonal anti-β-III tubulin (1:300; Sigma-Aldrich; St. Louis, MO, USA), rabbit polyclonal anti-cleaved-caspase-3-Asp 175 (1:300; Cell Signaling; Danvers, MA, USA), polyclonal goat anti-NTSR1 (1:50; Santa Cruz Biotechnology Inc.; Dallas TX, USA), and mouse monoclonal anti-TH (1:1000; Sigma- Aldrich; St. Louis, MO, USA). We used as the corresponding secondary antibodies Alexa Fluor 488 chicken anti-rabbit H + L IgG (1: 300; Invitrogen Molecular Probes; Eugene, Oregon, USA), Alexa Fluor 488 chicken anti-goat H + L IgG (1: 300; Invitrogen Molecular Probes; Eugene, Oregon, USA) and Texas Red horse anti-mouse H + L IgG (1: 900; Vector Laboratories; Burlingame, CA, USA). For negative controls, immunostaining was performed in the absence of the primary antibody and replacing it by the same IgG subclass. Some slices were incubated with Hoechst (Sigma- Aldrich; St. Louis, MO, USA) to stain cell nuclei. After washing with PBS, the slices were mounted on glass slides using VECTASHIELD (Vector Laboratories; Burlingame, CA, USA).

The β-sheet conformation of α-synuclein was detected in TH-immunolabeled slices counterstained with 0.05% Thioflavin T in 60% ethanol (Sigma- Aldrich; St. Louis, MO, USA) for 8 min, followed by 5 washes with 70% ethanol and MilliQ water as described elsewhere [89]. Some TH-immunolabeled slices were counterstained with a 0.0001% Fluoro Jade-C (F-J C) solution (Sigma-Aldrich; St. Louis, MO, USA) for 10 min to show neurodegeneration, as described previously [63].

An SP8 confocal microscope (Leica TCS SPE; Heidelberg, Germany) was used to analyze the double immunofluorescence at excitation-emission wavelengths of 358–461 nm (Hoechst), 488–522 nm (Alexa 488), and 568–635 nm (Texas Red). Serial 1-μm optical sections were also obtained in the Z-series (scanning rate of 600 Hz). LAS AF software (Leica Application Suite; Leica Microsystems; Nussloch, Germany) was used to process the images. The immunofluorescence area density (IFAD) for the double fluorescence assays was measured by ImageJ software v.1.46r (The National Institutes of Health; Bethesda, MD) in the injured and control sides of three anatomic levels along the SNpc per rat (*n* = 3 independent rats per group and time). The mean value calculated from the quantification in the three levels per nucleus and per rat was the final measurement.

### Densitometry and neuron counting

The mean intensity of TH (+) branches was measured in the injured and control sides in six anatomic levels along the *substantia nigra pars reticulata* (SNpr) and the striatum per rat. Background intensity was excluded from the immunohistochemically stained area. TH (+) neurons were counted in 8 slices of the SNpc (2 caudal, 4 medial and 2 rostral) per rat, as described previously [31, 68]. The total number of rats was three per every time point for the BSSG group, and six for the UT and mock groups; the rats of the latter group belonged to days 15 and 120 after the DMSO injection since there was no statistically significant difference when compared with the UT group. The immunohistochemical staining was analyzed with a Leica DMIRE2 microscope using the objectives 20x (SNpc) and 5x (striatum). Images were digitized with a DC300F camera (Leica; Nussloch, Germany). ImageJ software v.1.46r was used to measure the total area density of α-synuclein aggregates and TH (+) fibers in the SNpc, and optical density in the striatum. Fiji, an Image J complement, was used for color decomposition from the double staining of β-Gal staining with TH, GFAP, or Iba1 immunohistochemistry.

### Golgi-Cox staining

Rats were deeply anesthetized using sodium pentobarbital (75 mg/kg, i.p.) to perform euthanasia and perfused intracardially (0.9% saline solution). Brains were collected and stained by the modified Golgi-Cox method described previously [9, 23]. After storing in the dark for 14 days in the Golgi-Cox solution, and another three more days in 30% sucrose (wt/vol), brains were sectioned into 200-μm thick slides using a vibratome (Campden Instrument, MA752; Leicester, UK). Coronal sections were collected on clean gelatin-coated microscope slides. Staining was developed by using ammonium hydroxide for 30 min, followed by 30 min in Kodak Film Fixer. After washing and dehydrating, slices were cleared in successive baths of 50% (1 min), 70% (1 min), 95% (1 min), and 100% (5 min) alcohol, and in a xylene solution for 15 min. Then, slices were mounted in glass coverslips using a balsam resinous medium [27].

### Dendritic spines number and spine morphology analysis

MSNs from the dorsal striatum (Bregma, 1.7 mm to 0.2 mm, plates 11–17 of Paxinos and Watson Atlas, 1998) were identified through their soma size, dendritic extensions, and numerous dendritic spines, by a trained observer who was blind to the experimental conditions. A total of 240 neurons were analyzed; five neurons per hemisphere (injured and control sides) for each time in the three groups (*n* = 4–6 independent rats per group).

Dendritic spine density was quantified in each neuron along a 30-μm segment of distal dendrites at 1000x magnification and expressed as the number of spines/10 μm (DMLS Leica Microscope) [23]. The different spine shapes were counted in the same dendritic segments but at 2000x magnification. One hundred spines were classified according to the shape of their head and neck into five groups: mushroom (prominent and much higher diameter of head than the diameter of a well-identified neck), thin (the spine length longer than the neck diameter, and the diameters of the head and neck similar), stubby (wide spines with the neck diameter identical to the total length of the spine), bifurcated/branched (spines with two heads), multi-headed (spines with three or more heads) and unclassified spines (inconsistent with any of the previous criteria, less than 1%) [5, 73].

### Statistical analysis

Data were presented as the mean value ± the standard error of the mean (S.E.M.). Statistical analyses were performed with SigmaPlot 12.0. Intergroup differences were evaluated by bidirectional analysis of variance (2-way ANOVA), followed by Bonferroni post-hoc comparisons. For correlation analysis, Pearson’s correlation coefficient and subsequent linear regression were determined. Statistical difference was considered at *p* < 0.05.

## Results

### Unilateral intranigral BSSG administration triggers progressive aggregation and intracerebral spreading of pathological α-synuclein

**Fig. 1:**
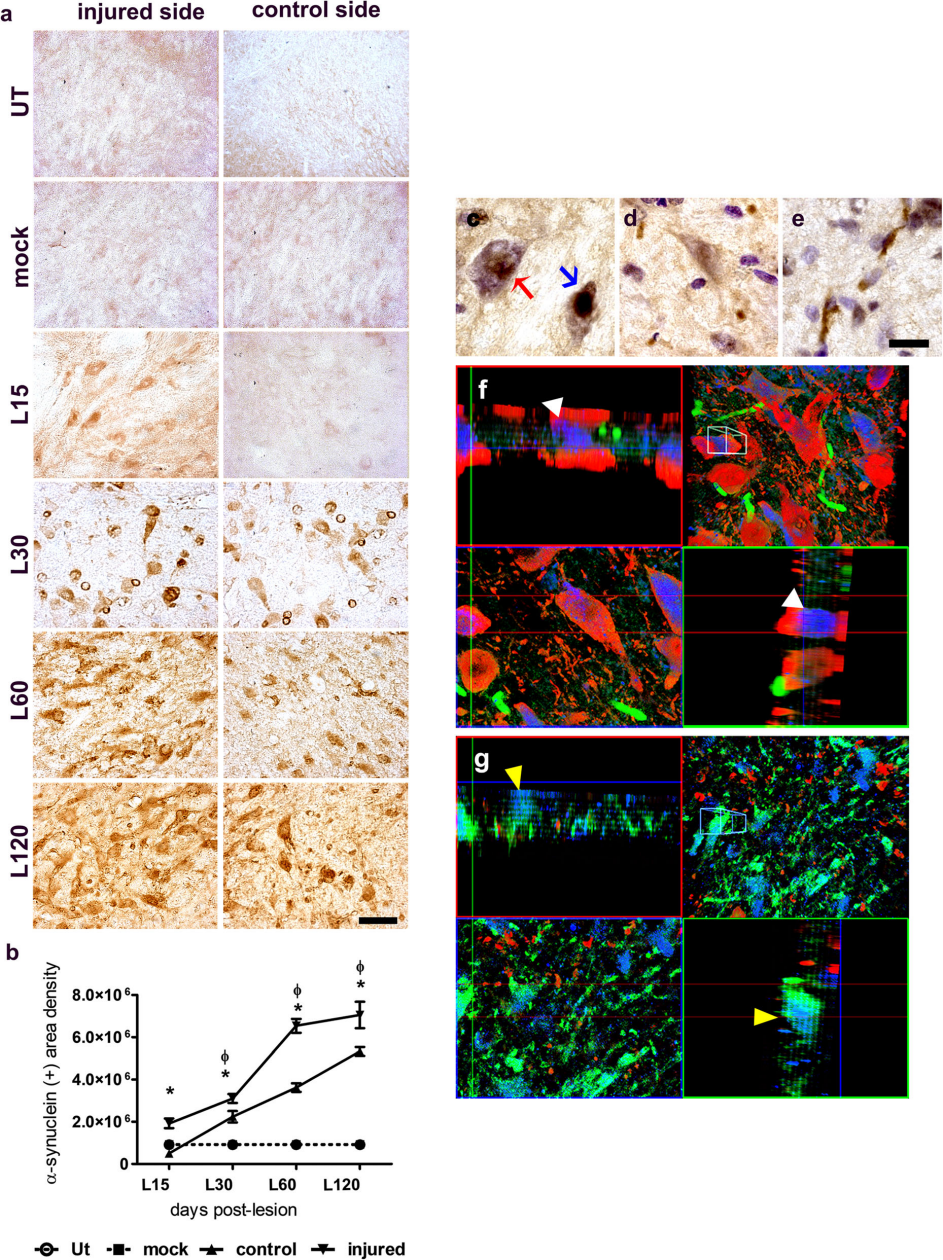
A single unilateral intranigral BSSG administration triggers progressive bilateral α-synuclein aggregates in the SNpc. **a** Representative micrographs of α-synuclein immunostaining. The scale bar = 50 μm is common for all micrographs. **b** Graph showing α-synuclein (+) area density of panel **a**. The values stand for the mean ± S.E.M. calculated from the measurements in three anatomical levels. *n* = 3 independent rats per each time in the BSSG group. The dashed lines correspond to the mock group (black square; *n* = 6 independent rats) and UT group (white circle; *n* = 6 independent rats). Φ,*, significant when BSSG group compared with the control groups. Two-way ANOVA, Bonferroni post-test. *p* < 0.05. **c** Amplifications showing α-synuclein aggregation patterns with diffuse (red arrow), and condensed (blue arrow) structures revealed by Nissl counterstaining, **d** dot-like structures and **e** Lewy neurites-like structures. The scale bar = 15 μm is common for all micrographs. **f**, **g** Double immunofluorescence against α-synuclein (blue) and TH (red) with Thioflavin-T counterstaining (green). Representative orthogonal projections from a 1-μm z-confocal optical section are the top left and bottom right panels. The top right panels are the integrated image, and the bottom left panels are a horizontal optical Z-section. **f** White arrowheads show α-synuclein aggregation within dopaminergic neurons. **g** Yellow arrowheads indicate colocalization of α-synuclein aggregation with Thioflavin T

BSSG caused a progressive and significant increase in pathological α-synuclein immunoreactivity in the SNpc of both sides, as compared with the mock group (Fig. 1a,b). The difference was significant (*p* < 0.05) from day 15 to 120 after the lesion in the injured SNpc, and from day 30 to 120 in the control SNpc (*p* < 0.01). The α-synuclein immunoreactivity showed different aggregation patterns [82], including diffuse and condensed staining (Lewy body-like aggregates; Fig. 1c), dot-like structures (Lewy dots; Fig. 1d), and thread-like structures (Lewy neurites; Fig. 1e). In the last two time points of the study, we identified TH (+) cells containing α-synuclein immunoreactivity without Thioflavin-T staining (Fig. 1f), suggesting the presence of soluble α-synuclein in dopaminergic neurons. Also, TH (−) cells with α-synuclein immunoreactivity and Thioflavin-T staining (Fig. 1g), suggested the presence of insoluble α-synuclein aggregates in degenerated neurons or other neuronal types.

**Fig. 2:**
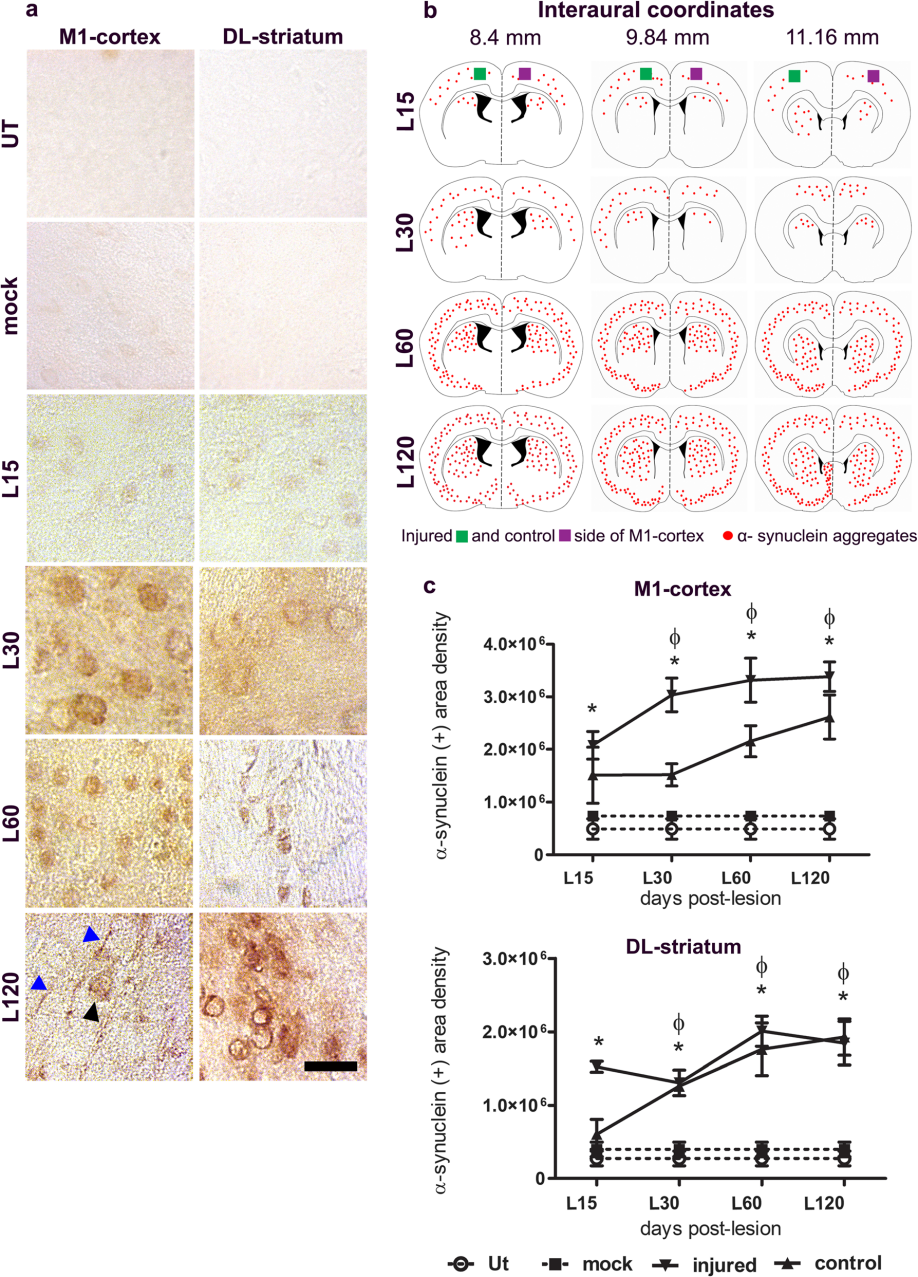
A single unilateral intranigral BSSG administration triggers progressive and bilateral pathological α-synuclein aggregates in the M1-cortex and DL-striatum. **a** Representative micrographs of pathological α-synuclein immunostaining in the injured side at the time displayed at the left in every row, evidencing cytoplasmic (black arrowhead) and dendritic (blue arrowhead) aggregation. The scale bar = 15 μm is common for all micrographs. **b** Schematic representation of coronal slices at three different levels showing the distribution of pathological α-synuclein over time. **c** Graphs showing the α-synuclein (+) area density on the injured and control sides of the M1-cortex and DL-striatum according to classification in Paxinos and Watson Atlas, 1998. The values are the mean ± S.E.M. calculated from the measurements in three anatomical levels. *n* = 3 independent rats per each time in the BSSG group. The dashed lines correspond to the mock group (black square; *n* = 6 independent rats) and UT group (white circle; *n* = 6 independent rats). Φ,*, significant when BSSG group compared with the control groups. Two-way ANOVA, Bonferroni post-test. *p* < 0.05

Besides, a progressive, bilateral and significant increase in the number of pathological α-synuclein aggregates was detected in the M1-cortex (*p* < 0.01) and DL-striatum (*p* < 0.001) (Fig. 2a-c), showing a similar time course to that in the SNpc. Intracellular aggregates of α-synuclein were observed in the striatum, whereas Lewy-neurite-like structures and intracellular aggregates were both observed in the M1-cortex (Fig. 2a,b). At the end of the study (120 days post-lesion), pathological α-synuclein aggregates were present in other midbrain nuclei (Online Resource 2) and other brain regions (Online Resource 3), suggesting propagation of pathological α-synuclein (Fig. 2a,b). No similar phenotypes were present in the mock group.

In the SNpc, the α-synuclein immunoreactivity was also observed within the microglia cells, in close proximity to neuronal cytoplasmic α-synuclein immunoreactivity (Online Resource 4a), but not within activated astrocytes, which instead seemed surrounded by α-synuclein aggregates (Online Resource 4b).

### Unilateral BSSG administration causes a progressive and bilateral decrease of dopaminergic phenotype in the nigrostriatal pathway

**Fig. 3:**
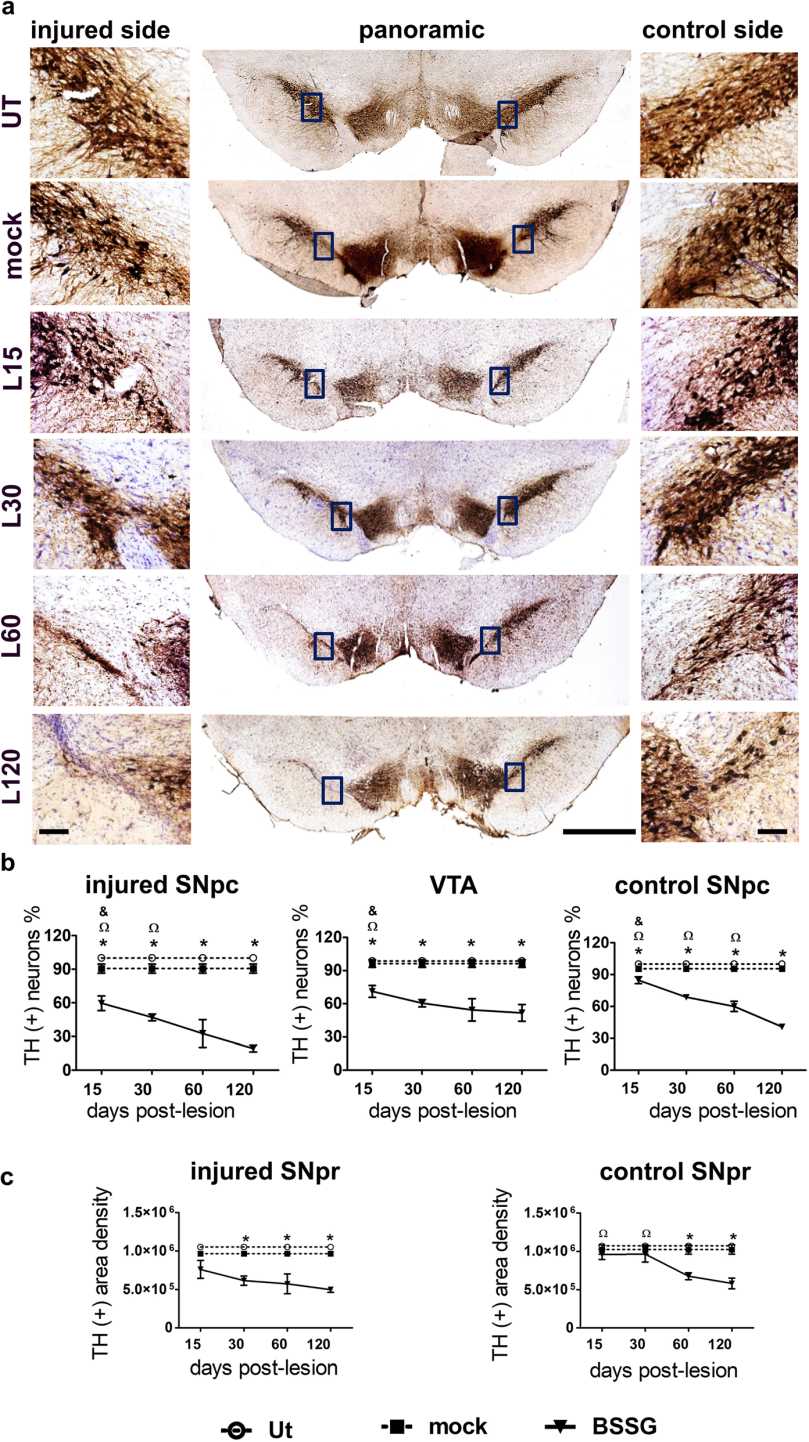
Unilateral intranigral administration of BSSG decreases TH (+) cells and their branches in midbrain nuclei. **a** Representative micrographs of TH immunohistochemical staining of mesencephalon slices. Times are displayed at the left margin in every row. The scale bars equal 1 mm for the panoramic views and 100 μm for the 20X zooms. **b** Graphs showing TH (+) cell counting in the injured SNpc, VTA, and control SNpc. **c** The area density of TH (+) arborization was measured in the injured and control SNPr. The values are the mean ± S.E.M. from three anatomical levels. *n* = 3 independent rats per time point in the BSSG group. The dashed lines correspond to the mock group (black squares; *n* = 6 independent rats) and the UT group (white circles; *n* = 6 independent rats). *, significant when compared with the control groups. When compared with the BSSG effect over time, the significance is marked with & vs. 60 and Ω vs. 120 days after the lesion. Two-way ANOVA, Bonferroni post-test. *p* < 0.05

BSSG significantly decreased the number of TH (+) cells in the SNpc of both sides (*p* < 0.001 for injured side, and *p* < 0.01 for the control one) and in the VTA (*p* < 0.05) from day 15 post-lesion, in comparison with the mock group (Fig. 3a,b). There was no statistical difference between the UT and the mock groups, although in the latter occurred a 10% decrease of TH (+) cells in the injured side (Fig. 3b). The maximum loss of TH (+) cells caused by BSSG was 71% (*p* < 0.001) in the injured SNpc, 55% (*p* < 0.001) in control SNpc, and 45% (*p* < 0.001) in the VTA, as compared with the mock group (Fig. 3b). The density of TH (+) fibers measured in the SNpr reached a maximum and significant decrease of 44% from day 30 on the injured side (*p* < 0.001), and 40% in the control side from day 60 (*p* < 0.001), as compared with the mock group (Fig. 3c).

**Fig. 4:**
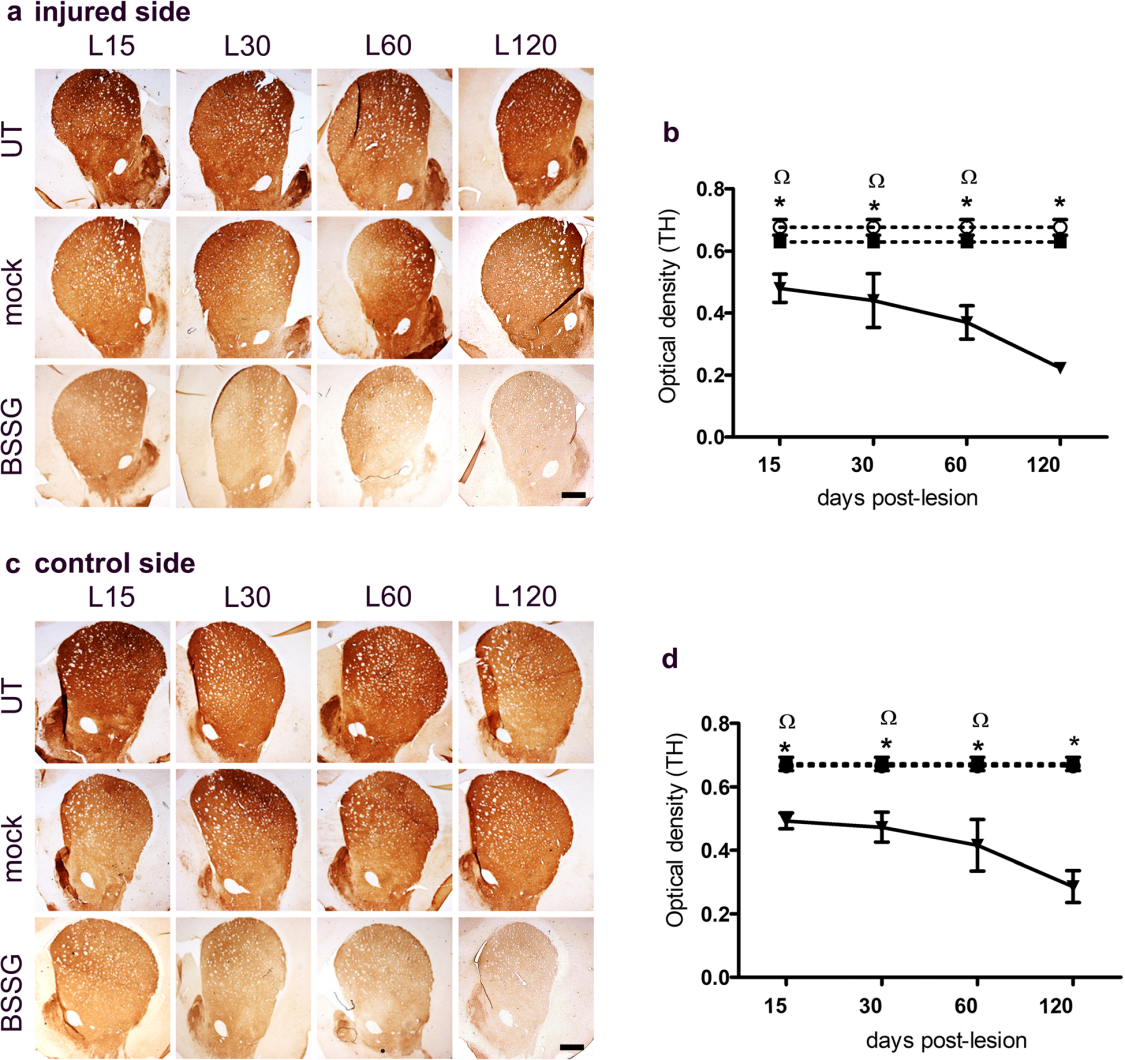
Unilateral intranigral administration of BSSG decreases TH (+) innervation in the striatum. Representative micrographs of TH immunohistochemical staining of **a** the injured and **b** control side (time points displayed at the bottom). The scale bars = 1 mm are common for all micrographs. The graphs show TH (+) optical density of the **b** injured and **d** control side. The values are the mean ± S.E.M. calculated from the measurements in three anatomical levels. *n* = 3 independent rats per each time in the BSSG group. The dashed lines correspond to the mock group (black squares; *n* = 6 independent rats) and the UT group (white circles; *n* = 6 independent rats). *, significant when compared with the control groups. When compared with the BSSG effect over time, the significance is marked with & vs. 60 and Ω vs. 120 days after the lesion. Two-way ANOVA, Bonferroni post-test. *p* < 0.05

BSSG also decreased the TH (+) area density in the striatum of both sides (Fig. 4a-d). Statistical significance was reached from day 15 on both sides (*p* < 0.001), when compared to the mock group (Fig. 4b,d). The maximum decrease in TH (+) density was 61% in the injured side and 57% in the control side of the striatum, as compared to the mock group (Fig. 4b,d). There was no statistical difference between the UT and the mock groups, which showed a 7% maximum decrease in the TH (+) area density only in the injured side.

### Unilateral BSSG administration causes a progressive and bilateral decrease of non-dopaminergic markers in the SNpc

**Fig. 5:**
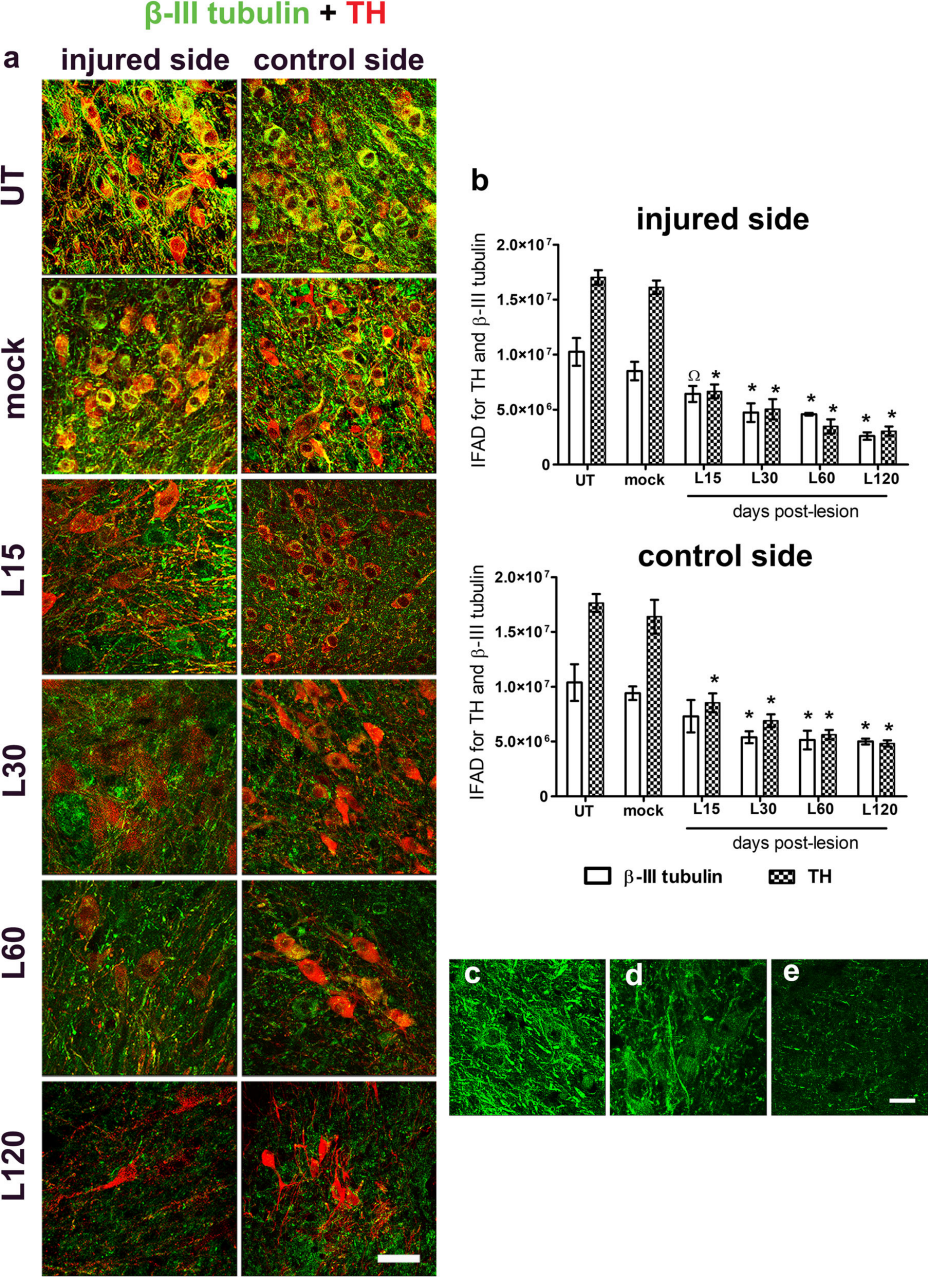
Unilateral intranigral administration of BSSG causes bilateral loss of neuronal cytoskeleton immunoreactivity. **a** Representative merged micrographs of double immunostaining against β-III tubulin (green) and TH (red) at the time displayed at the left margin in every row. **b** The graphs represent the IFAD for β-III tubulin and TH in the conditions of the panel **a**. The bars stand for the mean ± S.E.M. calculated from the measurements in three anatomical levels. *n* = 3 independent rats per time point in the BSSG group; *n* = 6 for Mock and UT groups. $, UT group compared with the mock group. *, Φ, BSSG group compared with the control groups. When compared with the BSSG effect over time, Ω vs. 120 days after the lesion. Two-way ANOVA, Bonferroni post-test. *p* < 0.05. Representative micrographs of cytoskeleton details in the SNpc of **c** mock condition and **d** injured condition at days 30 and **e** 120 after the BSSG injection. The scale bar = 50 μm is common for all micrographs in panel **a** and = 20 μm for the magnified images

BSSG progressively decreased the immunoreactivity of β-III tubulin, a neuronal cytoskeleton marker, in both the injured and control SNpc, with statistical significance from day 30 (*p* < 0.05), using the mock group as a control (Fig. 5a,b). As compared with the mock condition (Fig. 5a,c), profound disorganization of the neuronal cytoskeleton was observed from day 30 post-lesion (Fig. 5a,d) until the end of the study (Fig. 5a,e). The significant decrease in TH (+) IFAD (*p* < 0.001; Fig. 5b) was consistent with the loss of TH (+) cells and ramifications measured by immunohistochemistry (Fig. 3a-c).

**Fig. 6:**
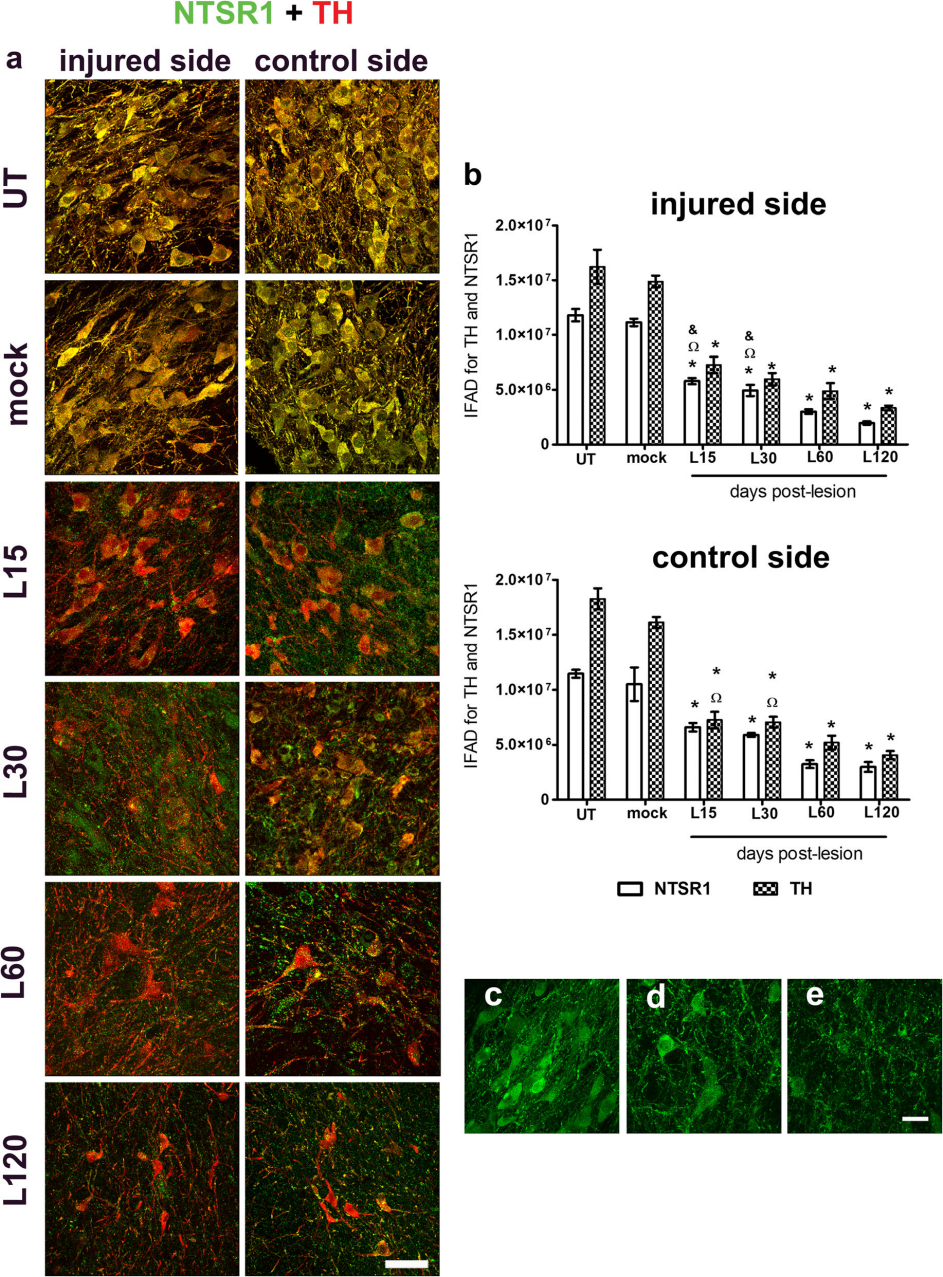
Unilateral intranigral administration of BSSG triggers bilateral loss of NTSR1 immunoreactivity. **a** Representative merged micrographs of double immunostaining against NTSR1 (green) and TH (red) at the time displayed at the left margin in every row. **b** Graphs showing IFAD of NTSR1 and TH in the conditions of the panel **a**. The bars stand for the mean ± S.E.M. calculated from the measurements in three anatomical levels. *n* = 3 independent rats per time point in the BSSG group; *n* = 6 for Mock and UT groups. *, BSSG group compared with the control groups. When compared with the BSSG effect over time, & vs. 60 and Ω vs. 120 days after the lesion. Two-way ANOVA, Bonferroni post-test. *p* < 0.05. Representative micrographs of NTSR1-immunoreactivity details in the SNpc of the c mock condition and **d** injured condition at days 30 and **e** 120 after the BSSG injection. The scale bar = 50 μm is common for all micrographs in panel **a** and = 20 μm for the magnified images

The double immunofluorescence analysis with TH and NTSR1 yielded similar results, except that the statistical significance occurred from day 15 after the lesion in the SNpc of both sides (*p* < 0.001 for injured side, and *p* < 0.05 for contralateral side), as compared with the mock group (Fig. 6a,b). A pronounced decline in NTSR1 immunoreactivity was observed from day 30 post-lesion (Fig. 6a,d) until the end of the study (Fig. 6a,e), in comparison with the mock condition (Fig. 6a,c).

### Unilateral BSSG administration causes bilateral neurodegeneration, senescence, and apoptosis in the SNpc

**Fig. 7:**
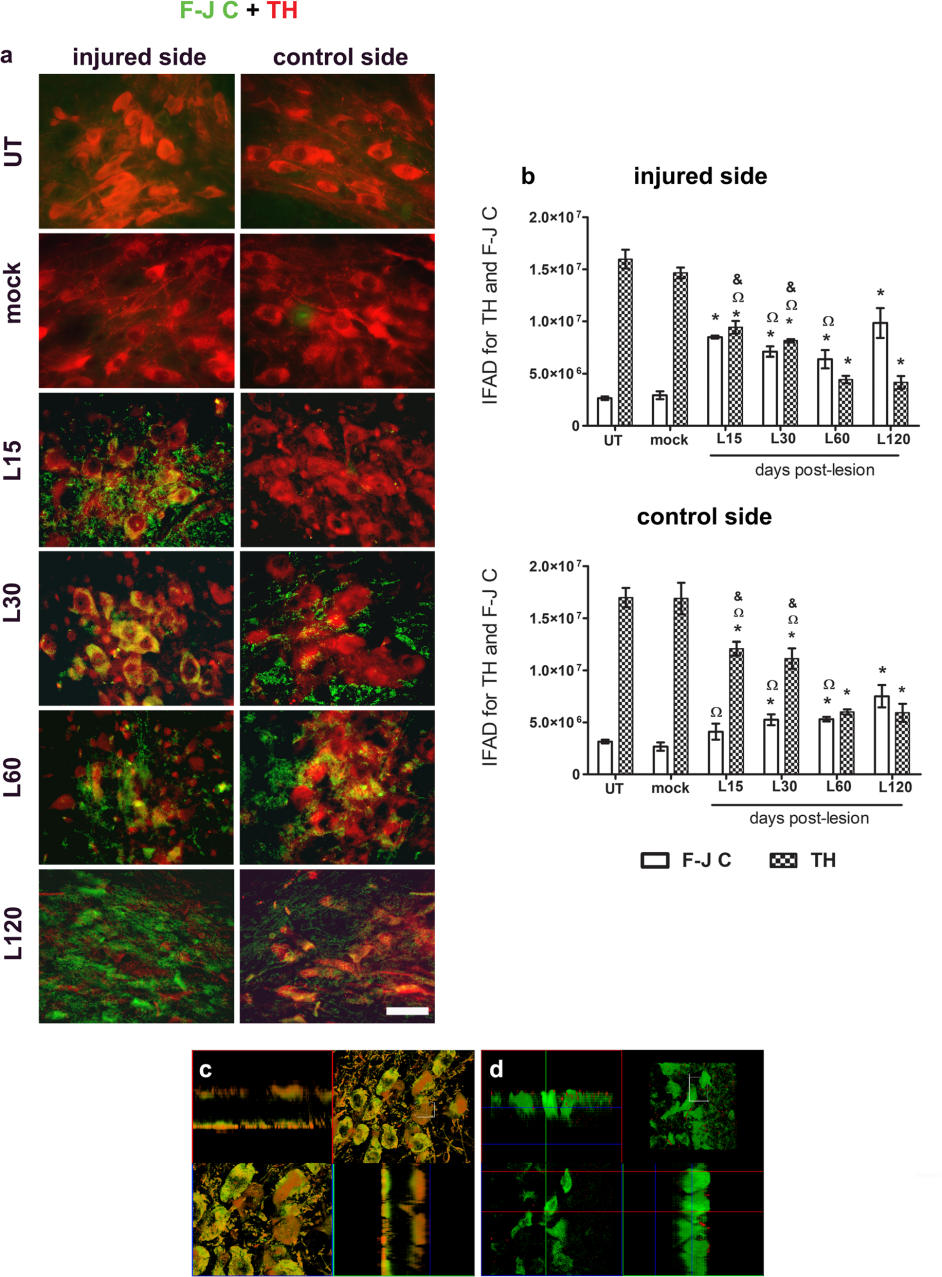
Unilateral intranigral administration of BSSG induces bilateral degeneration of the SNpc neurons. **a** Representative merged micrographs of slices stained with F-J C (green) and TH immunofluorescence (red) at the time displayed at the left margin in every row. The scale bar = 50 μm is common to all micrographs. **b** Graphs showing IFAD of F-J C and TH in the conditions of the panel **a**. The bars stand for the mean ± S.E.M. calculated from the measurements in three anatomical levels. *n* = 3 independent rats per time point in the BSSG group; *n* = 6 for Mock and UT groups. *, BSSG group compared with the control groups. When compared with the BSSG effect over time, & vs. 60 and Ω vs. 120 days after the lesion. Two-way ANOVA, Bonferroni post-test. *p* < 0.05. Analysis of confocal micrographs taken **c** at days 30 and **d** 120 after the BSSG administration. The top left and bottom right panels correspond to the orthogonal projections from 1 μm z-confocal optical sections. The right top panels are the integrated images, and the bottom left panels are a horizontal optical Z-sections

In the untreated and mock groups, the staining of F-J C, a neurodegeneration marker [63], was absent, and only TH (+) cells and ramifications were visible in the SNpc of both sides (Fig. 7a). In contrast, a bilateral increment of F-J C (+) IFAD occurred along with a decrease in TH (+) IFAD after intranigral BSSG administration (Fig. 7b). Colocalization of F-J C with TH fluorescence was observed on day 30 after the lesion on the injured side (Fig. 7a,c), whereas F-J C staining predominated on day 120 (Fig. 7a,d). F-J C and TH colocalization prevailed in the contralateral side up to the end of the study (Fig. 7a). In comparison with the mock group, the statistical difference was significant from day 15 in TH of both sides (*p* < 0.001 for injured side, and *p* < 0.05 for control side) and F-J C of the injured SNpc (*p* < 0.001), whereas F-J C was significant from day 30 in the control side (*p* < 0.01; Fig. 7b). These results show that the decline in the TH phenotype reflects dopaminergic neurodegeneration in the SNpc of both sides.

**Fig. 8:**
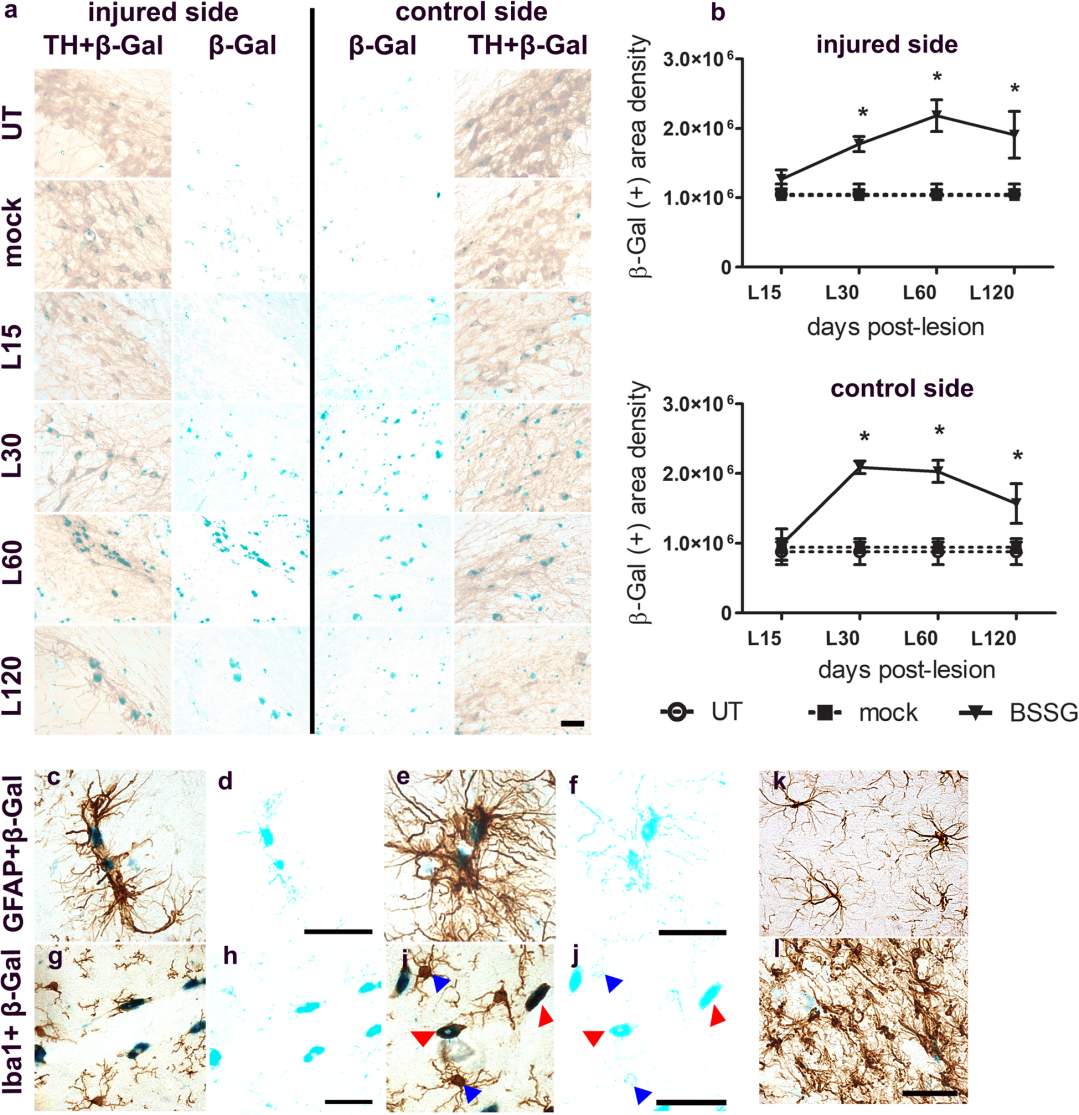
Unilateral intranigral administration of BSSG induces bilateral senescence in the SNpc. **a** Representative micrographs of double-stained slices with TH immunohistochemistry and β-Gal (TH + β-Gal) and their respective separated β-Gal staining (β-Gal). The opacity of TH immunoreactivity was decreased by 60% to allow visualization of β-Gal staining. The numbers at the left margin of every row indicate the day post-lesion. **b** The Graphs show β-Gal area density in the conditions of panel **a**. The bars stand for the mean ± S.E.M. calculated from the measurements in three anatomical levels. *n* = 3 independent rats per time point in the BSSG group; *n* = 6 for Mock and UT groups. *, BSSG group compared with the control groups. Two-way ANOVA, Bonferroni post-test. *p* < 0.05. Double stained slices with GFAP immunohistochemistry and β-Gal (**c**,**e**) and their respective separated β-Gal staining (**d**,**f**). Double stained slices with Iba1 immunohistochemistry and β-Gal (**g**,**i**) and their respective separated β-Gal staining (**h**,**j**). Red arrowhead shows ameboid microglia in senescence, and the blue arrowhead shows active microglia. **k** Astrocyte shape is illustrated in the conditions mock and **l** BSSG on day 60 after the lesion. The scale bar = 50 μm is common for all micrographs

β-Gal staining, a senescence marker [17], coincided with TH (+) cells since day 30 in the SNpc of both sides, and its area density was significantly higher (*p* < 0.05) than in the controls (Fig. 8a,b). This coincidence suggests that the senescence process participates in the BSSG-induced dopaminergic neurodegeneration. β-Gal staining was also observed in GFAP (+) astrocytes and Iba1 (+) microglia along the blood vessels (Fig. 8c,d,g,h), and in the SNpc parenchyma (Fig. 8e,f,i,j). Particularly, in microglia cells, β-Gal staining was observed on Iba1 (+) cells with ameboid shape, but not on ramified cells (Fig. 8f-j). Interestingly, degenerated astrocytes were observed on days 60 and 120 after the lesion when compared with the astrocytes of the mock condition at the same time (Fig. 8k,l).

**Fig. 9:**
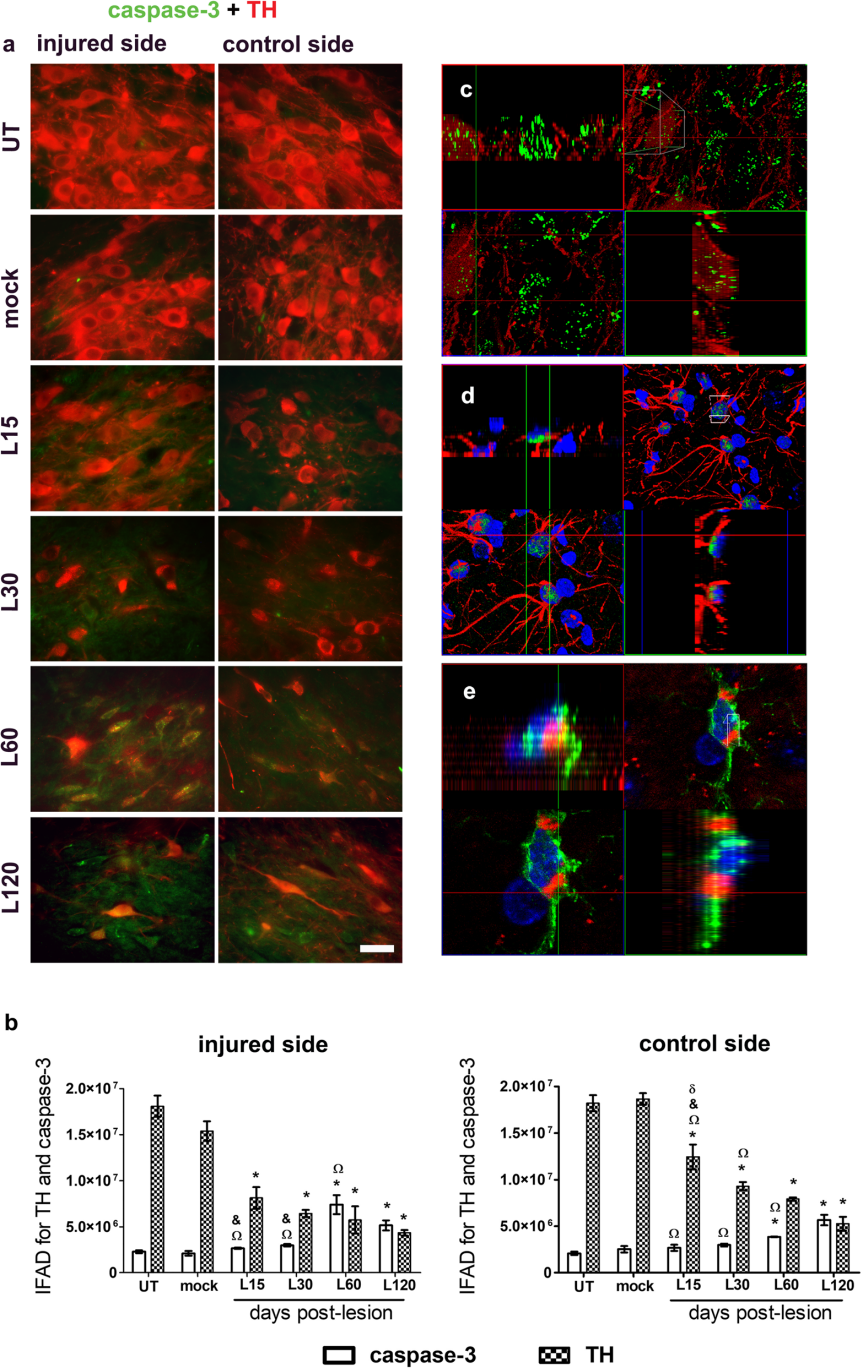
A single intranigral injection of BSSG triggers a bilateral and progressive increase of active caspase-3 immunoreactivity in the SNpc. **a** Representative merged micrographs of double immunostaining against active caspase-3 (green) and TH (red) at the time displayed at the left margin in every row. The scale bar = 50 μm is common for all micrographs. **b** The graphs show IFAD of the conditions in panel **a**. The bars stand for the mean ± S.E.M. calculated from the measurements in three anatomical levels. *n* = 3 independent rats per time point in the BSSG group; *n* = 6 for Mock and UT groups. *, BSSG group compared with the control groups. When compared with the BSSG effect over time, & vs. 60 and Ω vs. 120 days after the lesion. Two-way ANOVA, Bonferroni post-test. *p* < 0.05. **c** Confocal analysis of double immunostaining with cleaved caspase-3 (green) and TH (red); **d** cleaved caspase-3 (green), GFAP (red) and nuclear counterstaining (blue); **e** Iba1 (green), cleaved caspase-3 (red) and nuclear counterstaining (blue). The top left and bottom right panels correspond to the orthogonal projections from 1-μm z-confocal optical sections. The top right panels are the integrated images, and the bottom left panels are horizontal optical Z-sections

Cleaved caspase-3 staining was absent in the control groups and appeared in the SNpc of both sides until day 60 after the lesion (Fig. 9a), following the FJ-C and β-Gal staining. In the last two times of the study, the cleaved caspase-3 IFAD was significantly higher (*p* < 0.05) than the controls (Fig. 9b). Cleaved caspase-3 fluorescence co-localized with TH (+) (Fig. 9c), GFAP (+) (Fig. 9d) and Iba1 (+) (Fig. 9e) cells.

### Unilateral BSSG administration elicits a bilateral decrease in dendritic spine density of the striatal medium spiny neurons

**Fig. 10:**
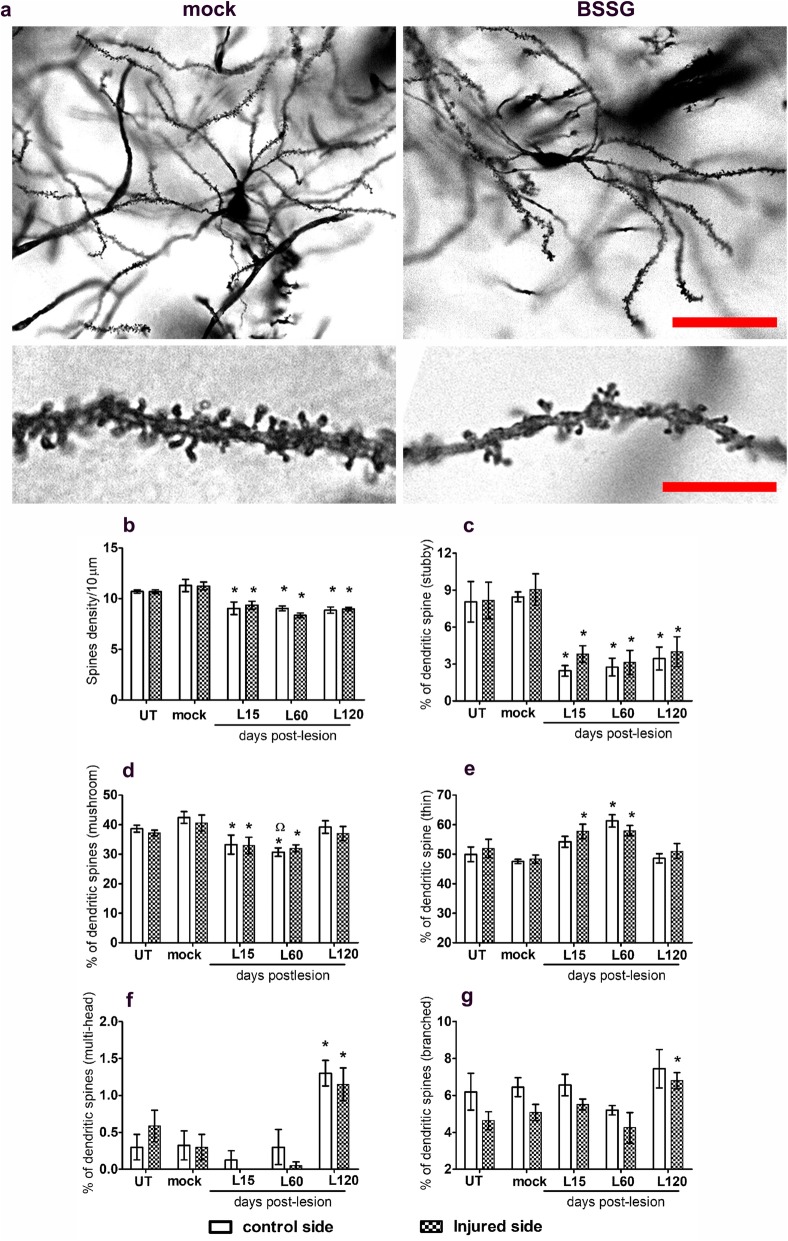
A single intranigral BSSG administration triggers bilateral morphologic changes and the density of dendritic spines in medium spiny neurons. **a** Representative micrographs showing Golgi–Cox-impregnated dendrites and spines of the mock and BSSG groups. The scale bar is 100 μm for panoramic views and 10 μm for amplifications. The graphs show the spine density (**b**) and the percentage of different spine types: stubby (**c**) mushroom (**d**), thin (**e**), multi-headed (**f**), and branched (**g**). The bars stand for the mean ± S.E.M. *n* = 4–6 independent rats per each group. *, BSSG group compared with the mock group. Two-way ANOVA, Bonferroni post-test. *p* < 0.05

The intranigral BSSG administration also caused atrophy of medium spiny neurons of both neostriatal nuclei (Fig. 10a), and a significant decrease in the dendritic spine density since day 15 post-lesion (*p* < 0.01 for injury side, and *p* < 0.001 for control side), as compared with the mock group (Fig. 10a,b). The effect on dendritic spines was differential and bilateral. The maximum decrease occurred in the stubby spines (70%; *p* < 0.01), followed by the mushroom spines (35%; *p* < 0.001) in the striatum of both sides (Fig. 10c,d). A significant increase was observed in thin spines (25%; *p* < 0.001) of the control side on day 60 post-lesion (Fig. 10e) as compared with the controls. A significant increase also occurred in multi-head spines (270%; *p* < 0.01) of both cerebral sides (Fig. 10f), and in branched spines (35%; *p* < 0.05) of the injured side at day 120 (Fig. 10g).

### Unilateral BSSG administration triggers motor and non-motor alterations

**Fig. 11:**
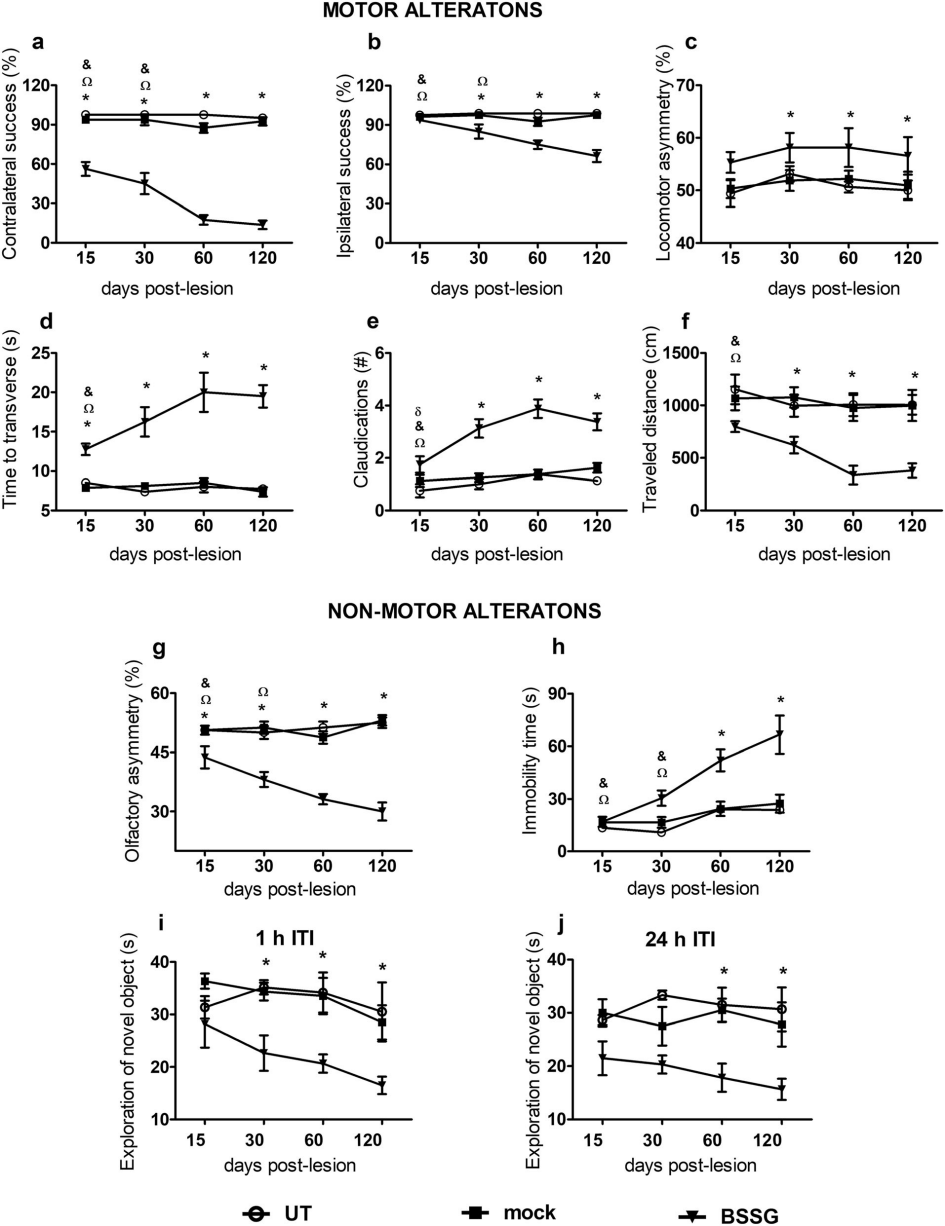
Unilateral intranigral administration of BSSG causes progressive motor and non-motor impairments. **a** Contralateral and **b** ipsilateral response to the vibrissae stimulation. **c** Asymmetry in the number of contacts of the forelimb paws on a transparent cylinder wall. **d** Time traveled and **e** claudications during displacement on a narrow beam. **f** Locomotor activity during the exploration of a new environment. **g** Asymmetry in discrimination of olfactory stimuli regularly distributed on each side of a corridor floor. **h** Depressive-like behavior evaluated by the immobility time in a swim tank. **i** Working and **j** episodic memory alteration evaluated by the exploration of the novel object in the open-field arena. *, BSSG group compared with the control groups. When compared with the BSSG effect over time, # vs. 30, & vs. 60 and Ω vs. 120 days after the injury. Each bar represents the mean ± S.E.M. from *n* = 8 rats per experimental group and time. Two-way ANOVA, Bonferroni post-test. *p* < 0.05

BSSG caused a progressive impairment in the motor and non-motor behavior evaluated with all the test sets, as compared with the mock group (Fig. 11), except in the locomotor asymmetry evaluated by the cylinder test and memory alteration evaluated by the NOR test (Fig. 11c,i,j). The first behavior impairments appeared from day 15, with the absence of contralateral motor response (*p* < 0.001; Fig. 11a), altered gait (*p* < 0.01; Fig. 11d), and olfactory asymmetry (*p* < 0.05: Fig. 11g). The second set of behavioral alterations appeared from day 30; these included the absence of motor response ipsilateral to the injured side (*p* < 0.01; Fig. 11b), postural instability (*p* < 0.001; Fig. 11e), locomotor asymmetry (*p* < 0.05; Fig. 11c), a decreased locomotor activity (*p* < 0.05; Fig. 11f) and a decrease in working memory (*p* < 0.05; Fig. 11i). Finally, on day 60 after the lesion, a statistical significance was observed in the depressive-like behavior (*p* < 0.01; Fig. 11h) and the episodic memory (*p* < 0.05; Fig. 11j). In agreement with the bilateral dopaminergic neurodegeneration, the vibrissae and cylinder tests revealed the development of bilateral sensorimotor affectation by the unilateral BSSG administration (Fig. 11a-c).

## Discussion

Our results show that a BSSG administration into the SNpc causes progressive aggregation of endogenous α-synuclein into Lewy body-like structures in the recipient side, and a posterior spreading to the contralateral side and other brain nuclei. Firstly, α-synuclein immunoreactivity appears within cells that are TH (+) and Thioflavin (−), and then in TH (−) cells with Thioflavin staining, suggesting that the misfolding of α-synuclein starts in dopaminergic neurons that later on degenerate. This evidence supports the proposal that the pathological α-synuclein aggregates cause dopaminergic neurodegeneration. However, this suggestion is not sustained by the high grade of correlation between the increase in pathological α-synuclein immunoreactivity and the decreased percentage of TH (+) neurons in the ipsilateral side to the injection, since those events coincide (*p* < 0.01; Online Resource 5a). On the contrary, the appearance of Lewy body-like synuclein aggregation, followed by the dopaminergic neurodegeneration in the control untreated side (*p* < 0.010; Online Resource 5b), supports the spreading and toxicity of α-synuclein aggregates. This assertion is further supported by the presence of pathological α-synuclein aggregates in cerebral nuclei associated with their respective motor and non-motor alterations. The presence of those aggregates in the striatum was associated with sensorimotor impairments, in the olfactory bulb with hyposmia, in the hippocampus with memory alteration, and in VTA with TH (+) cell loss and the development of depressive-like behavior. Those areas are anatomically and physiologically interconnected, thus explaining the spreading of α-synuclein pathology in a prion-like manner [3, 19, 35, 37, 55, 81].

The mechanisms of α-synuclein aggregation are still in the characterization process, including those underlying the genetic causes [10]. The BSSG neurotoxin might induce α-synuclein aggregation by modification of one of its multiple posttranslational mechanisms, which include phosphorylation, oxidation, acetylation, ubiquitination, glycation, glycosylation, nitration, and proteolysis [10]. Considering that BSSG is a steryl glucoside, it might be incorporated by glycation to α-synuclein, thus changing the protein charge and structure. Those modifications can lead to the misfolding of α-synuclein, hence, altering its interaction with other proteins and lipids, and the overall protein hydrophobicity [10, 79]. Further studies are needed to clarify the aggregation mechanism of α-synuclein, and the stereotaxic BSSG model could contribute to solve this question and develop inhibitory or disruptive therapies against the formation of α-synuclein aggregates [59].

The spreading mechanism of α-synuclein pathology is not currently fully resolved [81]. The most accepted mechanism involves the packing of toxic α-synuclein into exosomes and their transport across anatomical pathways of communication among brain regions, where the axon terminals release the toxic α-synuclein to be taken up by local cells. Therefore, the propagation pathway of pathological α-synuclein aggregates from the SNpc to brain nuclei of the ipsilateral side can be the dopaminergic projecting axons [1, 2, 14, 22], as suggested by previous works using PFFs [46, 56]. The vast web of interhemispheric connections mainly achieved by the four commissural systems [71] can transfer α-synuclein aggregates to the other brain hemisphere. Notably, the crossed connections of the *substantia nigra* [25, 58] and striatum [44] in the rat can also participate in α-synuclein aggregates transfer to those nuclei of the opposite hemisphere. The stereotaxic BSSG model represents a useful tool to identify the spreading mechanism of pathological α-synuclein and assay new exosome-based therapies [62].

A previous study has shown that the chronic oral administration of BSSG replicates α-synuclein aggregation, according to the Braak stages and the nigrostriatal dopaminergic neurodegeneration of PD [78]. However, the systemic presence of BSSG does not allow us to determinate whether α-synuclein aggregates cause the dopaminergic neurodegeneration or vice versa. A similar inconclusive outcome is also derived from the findings in the SNpc ipsilateral to the BSSG administration. In contrast, the results in the contralateral control SNpc show that α-synuclein aggregates preceded the loss of TH (+) cells, which also lost their immunoreactivity to β-III tubulin and NTSR1, and gradually gained staining to F-J C and β-Gal. Besides, an active caspase-3 immunoreactivity was present in the last two-time points of the study.

Altogether, these results suggest that pathological α-synuclein aggregates might induce the death of dopaminergic neurons by activating apoptosis and presumably senescence in the injured side as the correlation/regression analysis suggests (Online Resource 5c,e). However, the α-synuclein aggregates correlated with apoptosis (Online Resource 5d) but not with senescence in the untreated SNpc (Online Resource 5f). These results suggest that senescence might be triggered by an independent mechanism of pathological α-synuclein aggregates, for instance, the action of BSSG that only was present on the injured side. Functional studies in vitro are needed to clarify whether the pathological α-synuclein aggregates are the primary cause of senescence and apoptosis.

Several mechanisms have been proposed to explain the accumulation and toxicity of α-synuclein aggregates. Based on findings that lysosome is the main route for clearance of accumulated, misfolded, and toxic proteins, one possible mechanism is a dysfunction in the autophagy-lysosomal pathway [13, 33]. Besides, increasing evidence supports that the dysfunction of chaperone-mediated autophagy (CMA) promotes senesce [50]. BSSG might impair CMA, which is involved in the α-synuclein degradation [80], leading to toxic α-synuclein oligomers and senescence. In support of this suggestion is the correlation between the increased chaperone protein HSP70 levels and cell death after BSSG exposure in vitro [72], as occurs in the mutant LRRK2 knocking mouse model of PD with a similar chaperone protein [33]. The toxicity of α-synuclein aggregates could also be mediated by oxidative stress, which is another mechanism underlying cellular senescence [38] and apoptosis of dopaminergic neurons [31]. Mitochondrial disease cases and some forms of familial PD display mitochondrial dysfunction such as oxidative stress and deposition of pathological α-synuclein aggregates [20]; however, this possibility has not yet been explored after BSSG administration. BSSG-induced α-synuclein accumulation might also kill dopaminergic neurons through neuroinflammation [77], triggered by increased oxidative stress and direct activation of microglial cells [34, 83, 91]. In support of this mechanism, we found activated microglia cells apparently degrading neuronal cytoplasmic α-synuclein. This result is in agreement with recent findings that microglia and monocytes can take up free and exosome-associated α-synuclein oligomers resulting from age-dependent defects [6]. Another cell death mechanism of α-synuclein is excitotoxicity [17], and there is evidence that the exposure to BSSG triggers excitotoxicity mediated by the NMDA receptor in rat neocortex slices [85]. Therefore, the BSSG-induced pathological α-synuclein aggregates could cause the death of dopaminergic neurons by activating excitotoxicity.

Recently, it has been proposed that astrocyte and microglia could participate in the exosome-mediated clearance of α-synuclein [70]. However, the absence of α-synuclein immunoreactivity in GFAP (+) cells does not support such a role for astrocytes. In contrast, the presence of α-synuclein immunoreactivity in Iba1 (+) cells supports the involvement of microglia in the degradation of α-synuclein, as previously proposed [70]. Interestingly, the finding of β-Gal staining and active caspase-3 immunoreactivity in activated astrocyte and microglia suggests that these cells also die by senescence and apoptosis. This suggestion is supported by the presence of astrocytes with degenerated phenotype [36] and microglia cells with condensed ovoid shape. Dystrophic changes in dendritic spines of MSNs, which are the main target of dopamine axons [75], are invariably present in PD patients [37, 49] and experimental animals [60, 61, 67]. The loss of dopamine input, especially on stubby and mushroom spines, leads to an excessive corticostriatal glutamatergic excitation on MSNs that underlies motor impairments in PD [90]. In agreement with this physiological mechanism, the BSSG-induced dopaminergic denervation in the two striatal nuclei was associated with a significant decrease in stubby and mushroom dendritic spines of MSNs and the development of akinesia, bradykinesia, and uncoordinated gait. The increase in the percentage of thin, multi-headed, and branched spines, which are considered immature spines or in the maturation process [5], that occurred in the last time post-BSSG administration, might reflect an attempt to replenish the loss of mature spines (mushrooms). An alternative explanation for the dystrophy of dendritic spines of MSNs is the presence of α-synucleinopathy in the striatum. In PD patients, α-synuclein inclusions have been demonstrated in MSNs with neuritic changes in the striatum [51]. Accordingly, our results also show the development of aggregates and neurites-like structures of pathological α-synuclein in the two striatal nuclei. Together, the clinical and experimental findings suggest that α-synuclein aggregation can also induce the degeneration of striatal neurons, which is primarily reflected by a differential decrease in dendritic spine density.

The combined action of dopamine loss and α-synuclein toxicity in subcortical and cortical areas suggests neurodegeneration in these areas and the development of non-motor behaviors. For instance, hyposmia can result from the denervation of the nigrostriatal dopaminergic pathway, which projects directly to the olfactory bulb [3, 35], and the presence of pathological α-synuclein aggregates in this nucleus [3]. Likewise, pathological α-synuclein aggregates found in the hippocampus could impair both working and episodic memory, as observed with the BSSG systemic administration [78]. Depressive-like behavior can be explained by dopaminergic denervation of nucleus accumbens from VTA neurons, and the presence of pathological α-synuclein aggregates in those nuclei [55]. Moreover, nigral dopaminergic innervation of the forebrain, which is part of the mesolimbic system, is well documented in humans [76] and experimental animals [14, 48, 84]. Also, pathological α-synuclein aggregates were present in the forebrain after intranigral BSSG administration.

## Conclusion

Our results show that a single intranigral BSSG administration promotes the progressive appearance of pathological α-synuclein aggregates, and the death of dopaminergic neurons by senescence and then by apoptosis in the ipsilateral and contralateral SNpc. Thus, the resulting bilateral neurodegeneration of the nigrostriatal dopaminergic pathway and striatal MSNs elicited the motor deficits of parkinsonism. The prion-like spreading of toxic α-synuclein aggregates to the striatum, VTA, cerebral cortex, and olfactory bulb could also promote neurodegeneration in these areas, as suggested by the atrophy of striatal MSNs and the development of non-motor alterations. These features agree with the PD α-synucleinopathy phenotype, thus making the stereotaxic BSSG administration attractive for the identification of α-synucleinopathy spread mechanism and the validation of new therapies for PD.

## Supplementary information

**Additional file 1: Online Resource 1.** Illustration of experimental design. **a** Evaluation times of behavioral tests, immunohistochemistry, immunofluorescence, and Golgi-Cox staining as indicated by the symbols. The panel **b** shows a table with the number of animals used per assays every time point and group evaluated. Eight rats of each time point were evaluated with seven independent behavioral tests (*n* = 8 rats per experimental group and time).

**Additional file 2: Online Resource 2.** The ipsilateral and intranigral BSSG injection causes pathological α-synuclein propagation to the midbrain nuclei. Representative photomicrographs of (**a**) mock and (**b**) BSSG injured conditions showing α-synuclein aggregates in the red nucleus (1 and 2), SNpc (3 and 4), SNpr (5 and 6) and VTA (7). The scale bars = 1 mm for the panoramic views and 50 μm for magnifications.

**Additional file 3: Online Resource 3.** A single intranigral administration of BSSG causes pathological α-synuclein aggregates in different brain regions. Representative micrographs of α-synuclein immunohistochemistry in sagittal slices of (**a**) mock and (**b**) BSSG groups on day 120 after the lesion showing α-synuclein aggregates in the olfactory bulb (1), hippocampus (2), cortex (3), M1-cortex (4), locus coeruleus (5), *substantia nigra* (6) and striatum (7). The scale bars = 1 mm for the panoramic views and 100 μm for magnifications.

**Additional file 4: Online Resource 4.** Apparent phagocytosis of α-synuclein (+) neurons by microglia but not by astrocytes. Double immunofluorescence against α-synuclein (red) and Iba1 (green) or GFAP (green). Panels **a** and **c** are panoramic views. Panels **b** and **d** are orthogonal projections from 1-μm z-confocal optical sections that correspond to the top left and bottom right panels. The top right panels are the integrated image, and the bottom left panels are a horizontal optical Z-section. Yellow arrows show microglia with α-synuclein aggregation. The scale bar = 50 μm.

**Additional file 5: Online Resource 5.** Correlation analysis of α-synuclein area density with the survival percentage of dopaminergic neurons (**a** and **b**), immunofluorescence area density (IFAD) of active caspase-3 (**c** and **d**), and β-Gal(+) area density (**e** and **f**) in the injured and control SNpc. Pearson’s correlation coefficient and linear regression appear on the top of every graph. *p* < 0.05 was considered a statistically significant difference.

## Data Availability

All data generated or analyzed during this study are included in this published article (and its additional files).

## Abbreviations

2-way ANOVA: Bidirectional analysis of variance
ALS/PDC: Amyotrophic lateral sclerosis/parkinsonism dementia complex
BSSG: β-sitosterol β-D-glucoside
Cinvestav: Center for Research and Advanced Studies
CMA: Chaperone-mediated autophagy
DAB: 3′3-diaminobenzidine
DL: Dorsolateral
F-J C: Fluro-Jade C
SNCA: α-synuclein gene
GFAP: Glial fibrillary acidic protein
HRP: Horseradish peroxidase
i.p.: Intraperitoneal
Iba 1: Ionized calcium-binding adapter molecule 1
IFAD: Immunofluorescence area density
ITI: Inter-trial interval
M1: Primary motor cortex
MSN: Medium spiny neurons
NOR: Novel object recognition
NTSR1: Neurotensin receptor type 1
PBS: Phosphate-buffered saline solution
PD: Parkinson’s disease
PFF: α-synuclein preformed fibrils
RT: Room temperature
S.E.M.: Standard error of the mean
SNpc: The *substantia nigra pars compacta*
SNpr: *Substantia nigra pars reticulate*
TCS: Tissue collecting solution
TH: Tyrosine hydroxylase
UT: Untreated
VTA: Ventral tegmental area
