# Supplementary figures and images for: Unilateral intranigral administration of β-sitosterol β-D-glucoside triggers pathological α-synuclein spreading and bilateral nigrostriatal dopaminergic neurodegeneration in the rat
Source: Acta Neuropathol Commun. 2020 Apr 22;8:56. doi: 10.1186/s40478-020-00933-6 (PMC7178762; doi:10.1186/s40478-020-00933-6)

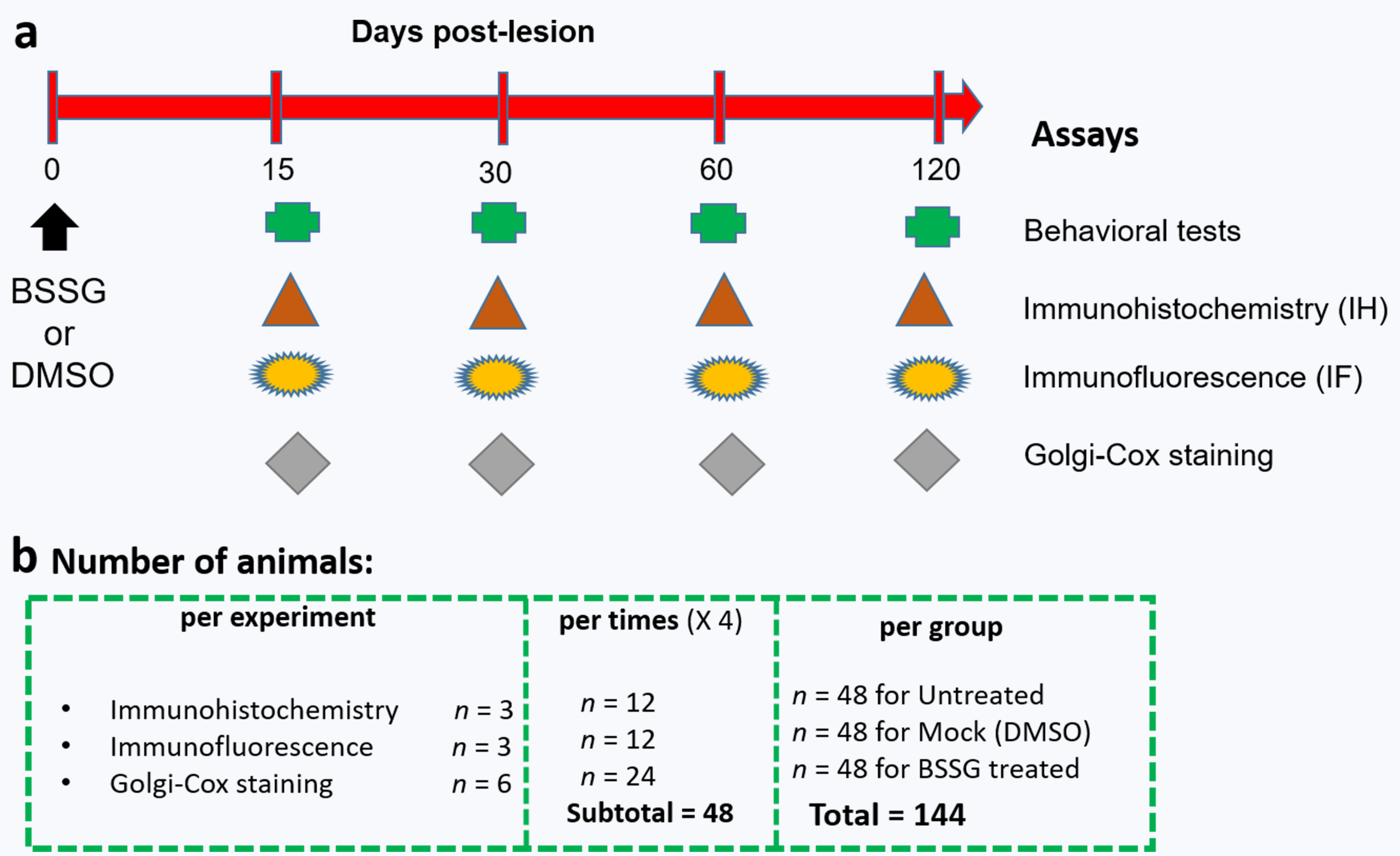

Supplement: Supplementary file 1 — Additional file 1: Online Resource 1. Illustration of experimental design. a Evaluation times of behavioral tests, immunohistochemistry, immunofluorescence, and Golgi-Cox staining as indicated by the symbols. The panel b shows a table with the number of animals used per assays every time point and group evaluated. Eight rats of each time point were evaluated with seven independent behavioral tests (n = 8 rats per experimental group and time). [file 40478_2020_933_MOESM1_ESM.tif]

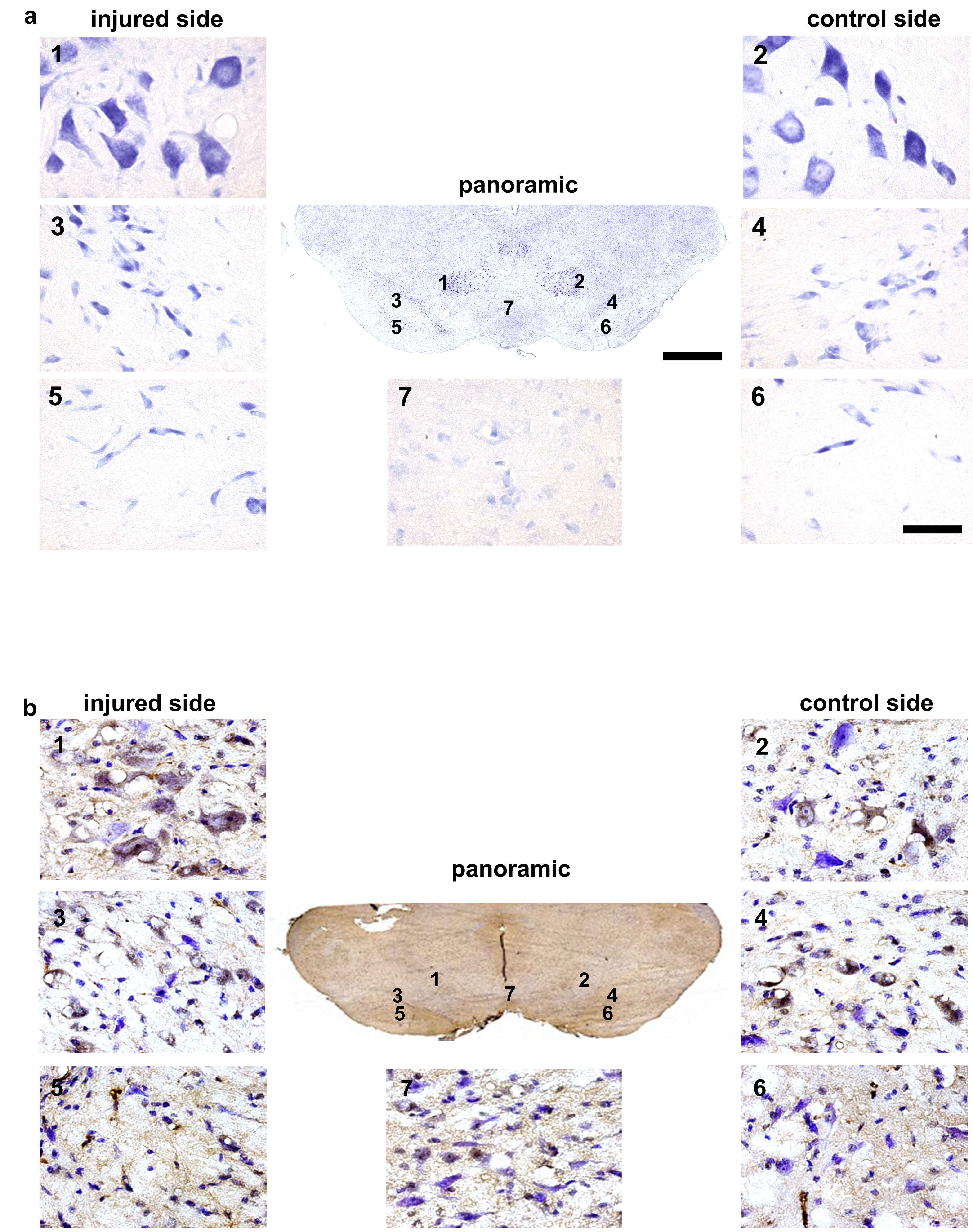

Supplement: Supplementary file 2 — Additional file 2: Online Resource 2. The ipsilateral and intranigral BSSG injection causes pathological α-synuclein propagation to the midbrain nuclei. Representative photomicrographs of (a) mock and (b) BSSG injured conditions showing α-synuclein aggregates in the red nucleus (1 and 2), SNpc (3 and 4), SNpr (5 and 6) and VTA (7). The scale bars = 1 mm for the panoramic views and 50 μm for magnifications. [file 40478_2020_933_MOESM2_ESM.tif]

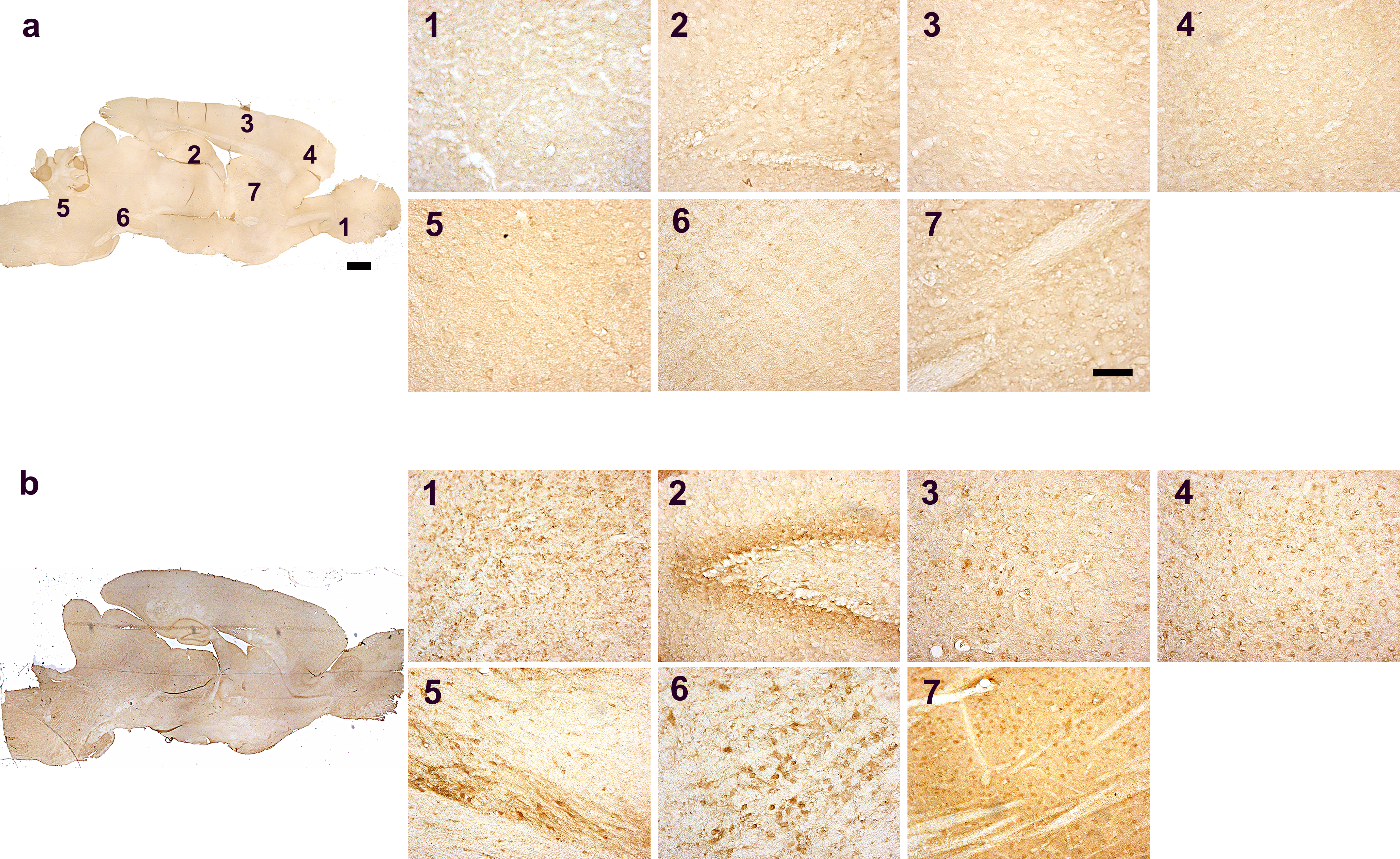

Supplement: Supplementary file 3 — Additional file 3: Online Resource 3. A single intranigral administration of BSSG causes pathological α-synuclein aggregates in different brain regions. Representative micrographs of α-synuclein immunohistochemistry in sagittal slices of (a) mock and (b) BSSG groups on day 120 after the lesion showing α-synuclein aggregates in the olfactory bulb (1), hippocampus (2), cortex (3), M1-cortex (4), locus coeruleus (5), substantia nigra (6) and striatum (7). The scale bars = 1 mm for the panoramic views and 100 μm for magnifications. [file 40478_2020_933_MOESM3_ESM.tif]

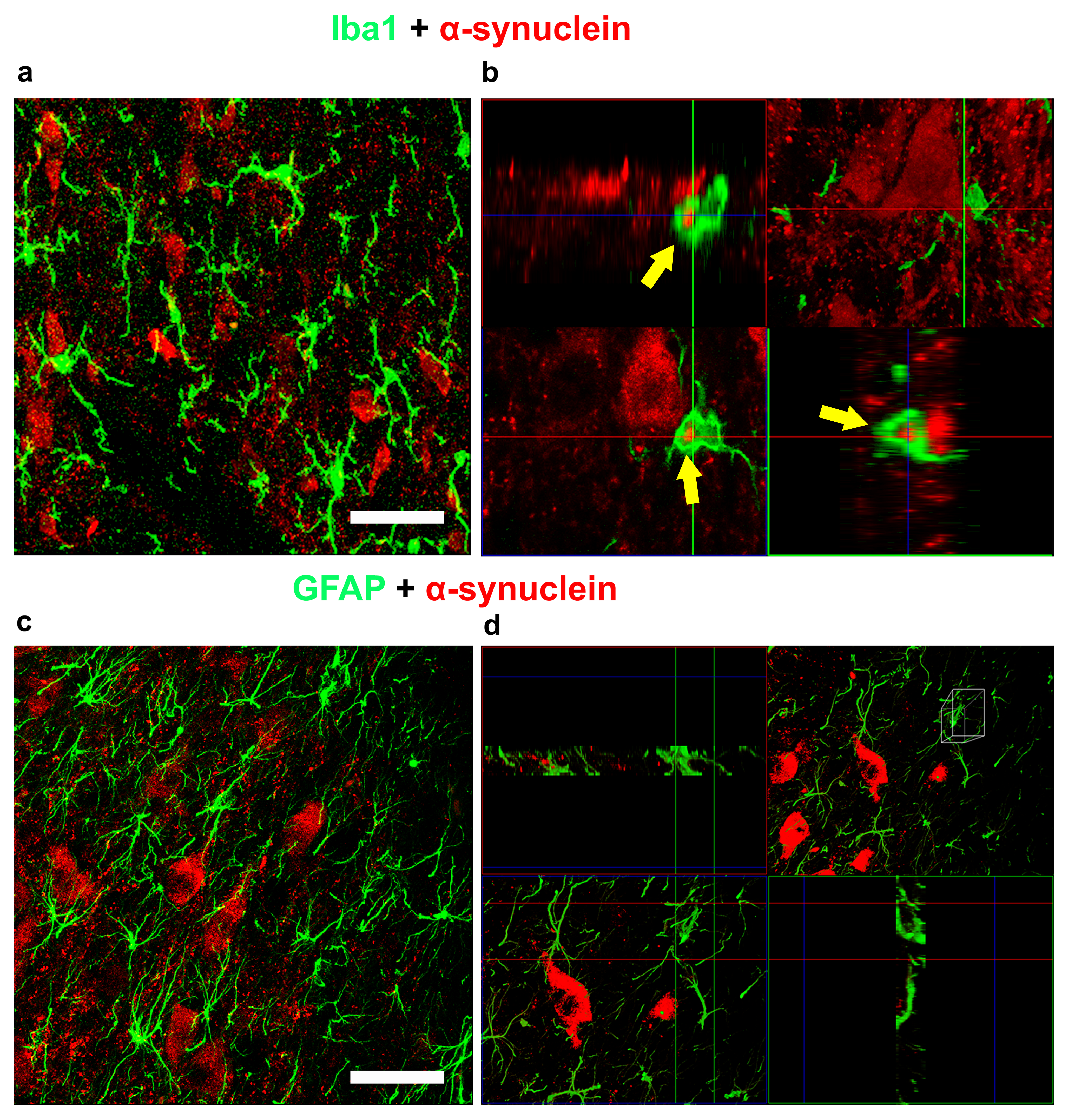

Supplement: Supplementary file 4 — Additional file 4: Online Resource 4. Apparent phagocytosis of α-synuclein (+) neurons by microglia but not by astrocytes. Double immunofluorescence against α-synuclein (red) and Iba1 (green) or GFAP (green). Panels a and c are panoramic views. Panels b and d are orthogonal projections from 1-μm z-confocal optical sections that correspond to the top left and bottom right panels. The top right panels are the integrated image, and the bottom left panels are a horizontal optical Z-section. Yellow arrows show microglia with α-synuclein aggregation. The scale bar = 50 μm. [file 40478_2020_933_MOESM4_ESM.tif]

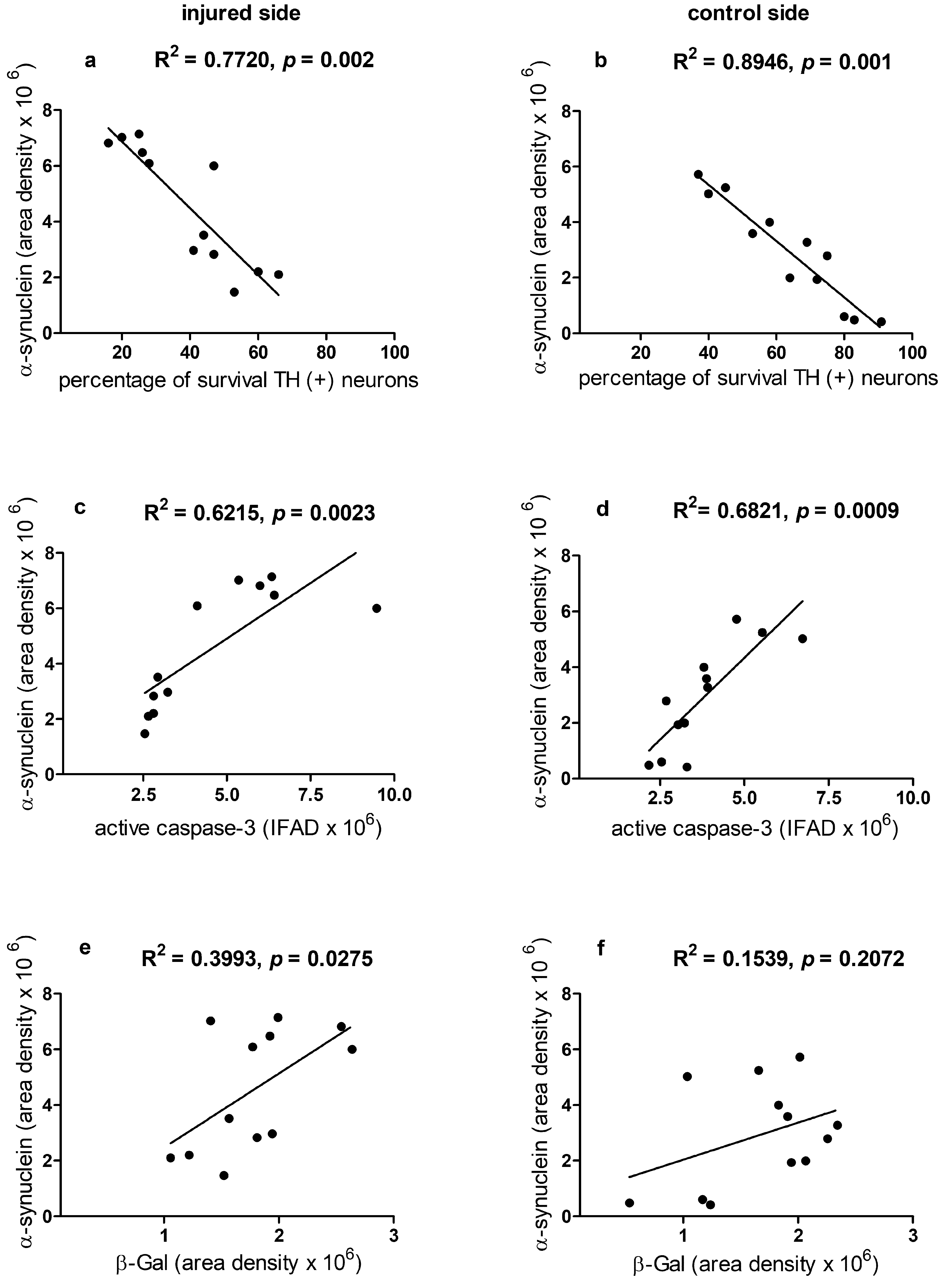

Supplement: Supplementary file 5 — Additional file 5: Online Resource 5. Correlation analysis of α-synuclein area density with the survival percentage of dopaminergic neurons (a and b), immunofluorescence area density (IFAD) of active caspase-3 (c and d), and β-Gal(+) area density (e and f) in the injured and control SNpc. Pearson’s correlation coefficient and linear regression appear on the top of every graph. p < 0.05 was considered a statistically significant difference. [file 40478_2020_933_MOESM5_ESM.tif]
